# Death by hanging: possibilities and limits of medico-legal evidence

**DOI:** 10.1007/s00414-026-03734-z

**Published:** 2026-02-21

**Authors:** Stefan Pollak, Markus Große Perdekamp, Annette Thierauf-Emberger

**Affiliations:** Institute of Forensic Medicine, Medical Center and Faculty of Medicine, University of Freiburg, Albertstraße 9, Freiburg, 79104 Germany

**Keywords:** Death by hanging, Diagnosis criteria, Signs of vitality

## Abstract

In forensic medicine, hanging is a frequent cause of non-natural death which may pose diagnostic problems on the investigator due to the manifold and sometimes ambiguous autopsy findings. The great variability of appearances potentially impedes the classification as to the manner of death (suicide, accident, homicide). The significance of some signs formerly considered to prove vital suspension could not always be confirmed in more recent studies. Therefore, traditional conception passed on from generation to generation must be critically questioned and, if necessary, replaced by evidence-based knowledge derived from systematic evaluations of relevant case material and laboratory tests, where appropriate. Bearing in mind the numerous possible misinterpretations, a dogmatic view must be avoided in favour of a cautious and holistic assessment. The review presented here provides an overview of problem areas based on the pertinent literature, photographic material of real cases and the authors’ professional experience.

## Introduction

For centuries, death by hanging has been a significant topic in forensic medicine. The relevant literature is abundant with case studies, surveys, overview papers, book chapters and monographs. Nevertheless, or precisely because of this, there seems to be still a need for reviews that summarize the current state of knowledge by reporting, analysing and discussing both established knowledge and current research results [1–20].

Only recently an Italian working group from Milan and Rome [21] published a comprehensive literature study with the aim of providing an overview of potentially significant signs and laboratory investigations suitable for the autoptic diagnosis of death by hanging. As the authors rightfully pointed out, there is an extraordinarily great variability in the reported frequency data of the signs associated with hanging deaths. This impedes the interpretation regarding the diagnostic value and practical usefulness of positive and negative findings.

According to our own experience, there is considerable uncertainty concerning the diagnostic criteria substantiating a hanging death or serving as proof of vitality. This might be due to the decreasing autopsy frequency in a group of cases which superficially are considered to be clear under the impression of the situation at the scene [22]. To correctly interpret morphological findings, it is extremely important to train the “diagnostic eye” [23, 24]. As the signs of asphyxia in general and especially those pointing to hanging may be scanty and discernible only for an experienced examiner, there is a high risk of misdiagnosis and incorrect interpretation. For this reason, the following survey is not primarily aimed at a detailed record of literature data but focuses more on the manifold morphological appearances and their – often doubtful – value regarding the post-mortem diagnosis of (vital) hanging.

Hanging is a subgroup of neck compression in which the force applied by a noose is derived from the gravitational drag of the victim’s body weight or part of it. With regard to the manner of death, a distinction is made between suicidal, accidental, homicidal and judicial hangings. Mostly, the suspension is brought about by a loop which tightens around the neck and thus applies pressure to its anatomical structures, especially the large blood vessels and the upper airways. There is a great variety of hanging devices including ropes, belts, cords, wires, strings, shoelaces, scarves, neckties, clotheslines, torn bed-sheets and many others. The upper end is usually attached to an elevated point such as a ceiling beam, a stair case railing, a wall hook, a doorknob, a bedpost or – outdoors – a large branch of a tree. The manifold scenarios encountered in forensic practice as well as epidemiologic data related to hanging deaths are outside the scope of this review which is focused on the evidential value of the findings seen in real cases.

In many countries, hanging is the most common method of suicide. Therefore, death by hanging is often exclusively associated with suicide thus ignoring the realistic alternatives of accidental death or homicide [25]. As Hofmann and Haberda rightly stated [26], the mere predominance of self-hangings may support the premature assumption of suicide. Especially examiners with limited experience are prone to underestimate the diagnostic challenges and possible pitfalls when investigating hanged victims [27].

Another problem is that the significance of some signs is not assessed uniformly throughout the published literature. An example of this are the intracutaneous haemorrhages often found on skin ridges between multiple turns of a noose. Many previous authors considered such extravasations as evidence of vitality, whereas others could demonstrate that a post-mortem formation is likewise possible [28–30].

The following compilation is intended to provide an overview of the current state of medico-legal knowledge concerning the autopsy findings in hanging deaths. In general, a holistic approach seems appropriate taking also into account the pathophysiological processes as well as the scene and the criminalistic aspects.

## Site and circumstances of the incident

Inspecting the scene in a hanging case is an integral element of the medico-legal assessment as to manner and cause of death. Whenever possible, the unchanged situation and the original state of the (still dressed) body should be surveyed and documented. This includes the height of suspension and the accessibility of the point, where the upper end of the hanging device is attached. Another important issue is the presence or absence of a support such as a chair or stepladder necessary to fix the ligature in an elevated location.

Many fatal hangings occur from low suspension points, as already a small part of the body weight can be sufficient to occlude the great neck vessels and/or the airways. Therefore, hanging is possible in different body positions such as in a standing, sitting, kneeling, lying or reclining posture [31–35]. On the other hand, a great drop height, for instance when jumping from a balcony or a bridge with the noose around the neck, may cause severe structural damage to the cervical spine and even decapitation. Some authors differentiate between ‘*complete hanging’* (in cases of full suspension without ground contact or any other support of the body) and *‘incomplete hanging’* (suspension with only part of the body weight acting on the noose) [36].

Most hanging devices form a noose, which may be ‘open’ (if the ends are not tied in a knot). In contrast, a slipknot tightens when tension is applied so that (nearly) the full circumference of the neck is constricted. A fixed noose has an immovable knot and usually causes a hanging mark rising to a gap at its highest point. In cases of hanging with dual or multiple loops, the skin between the ligature turns may be squeezed showing intradermal blood extravasations and/or blisters [30, 37]. There is a great variety of noose configurations including those consisting of circular turns and ascending ends which cross each other at the front or back of the neck. The loop may even imitate a pulley system [38].

According to the position of the knot, a distinction is made between *‘typical’* hangings (with the highest point of the ligature located on the nape of the neck or in the adjacent occipital region) and *‘atypical’* cases (with the knot positioned on the front of the neck or laterally). Most studies conclude that atypical hangings are more common. Apart from flexible hanging devices, the neck pressure can also be exerted by a solid object such as a branch fork, a furniture edge or a window frame.

In *complex suicides*, hanging is preceded or accompanied by at least one other method (e.g. wrist and/or neck cutting, stab wounds to the chest, drug intoxication or ingestion of caustics, self-inflicted gunshot injury) [39, 40]. In contrast, *‘complicated suicides’* are characterized by an unintentional secondary traumatization following the method primarily applied. This group includes fatalities due to unintended falls from a height, for instance when the hanging noose breaks [41].

The situation at the scene may give reconstructive hints on the course of events. So blood stains are possibly due to a failed hanging attempt followed by a fall to the ground causing a scalp laceration [27]. The distribution of blood stains from a previously inflicted injury may be indicative of the body’s movements during hanging [42]. If fingers are wedged between loop and neck, the ligature mark is interrupted by vertical or oblique impressions, whereas the interposed fingers may show blisters distal from the ligature groove [43]. The continuity of the hanging mark can also be interrupted by textiles, necklaces or strands of hair wedged between the noose and the neck.

**Fig. 1:**
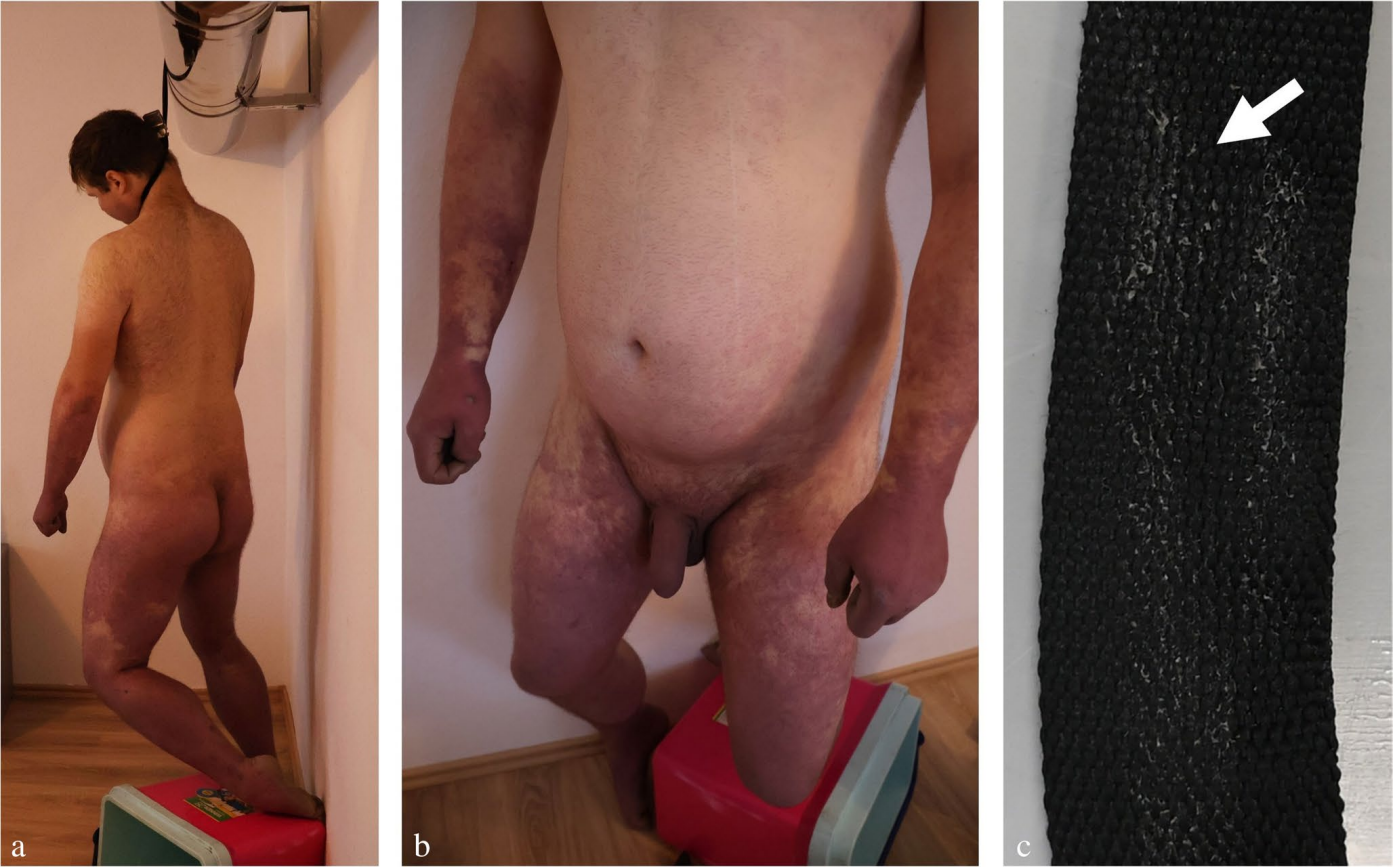
Autoerotic accident caused by hanging (**a**, **b**). The body is unclothed, the left foot is partly supported by the climbing aid (tipped-over plastic container). The post-mortem lividity is located on the limbs hanging down. (**c**) Ligature with abraded horny scales from the neck skin (arrow)

**Fig. 2:**
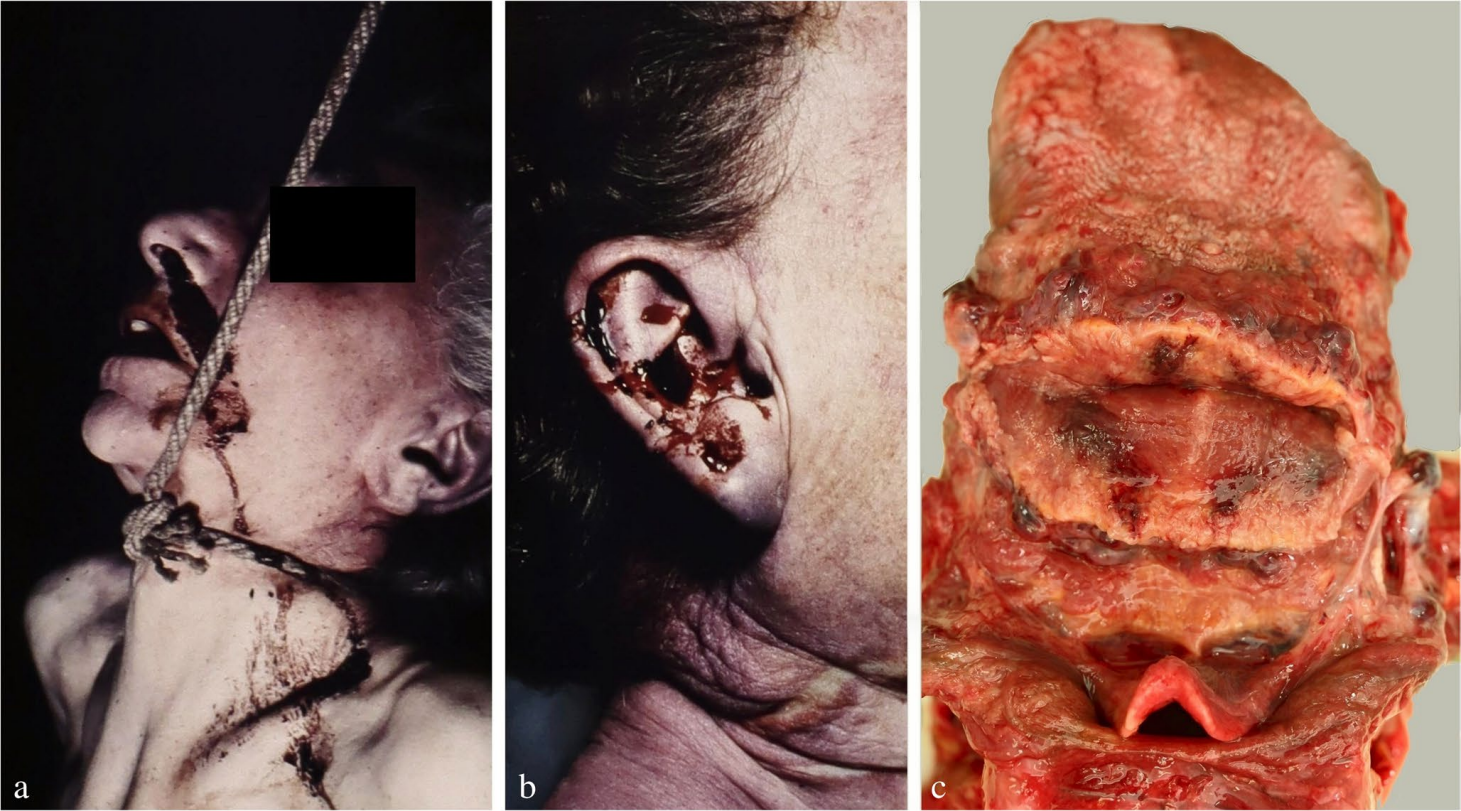
Bleeding from the nose (**a**) and the right external auditory canal (**b**) in suicidal hangings accompanied by venous congestion in the head area (cf [23]). (**c**) Base of the tongue with subepithelial haemorrhages (made visible by transverse incisions)

**Fig. 3:**
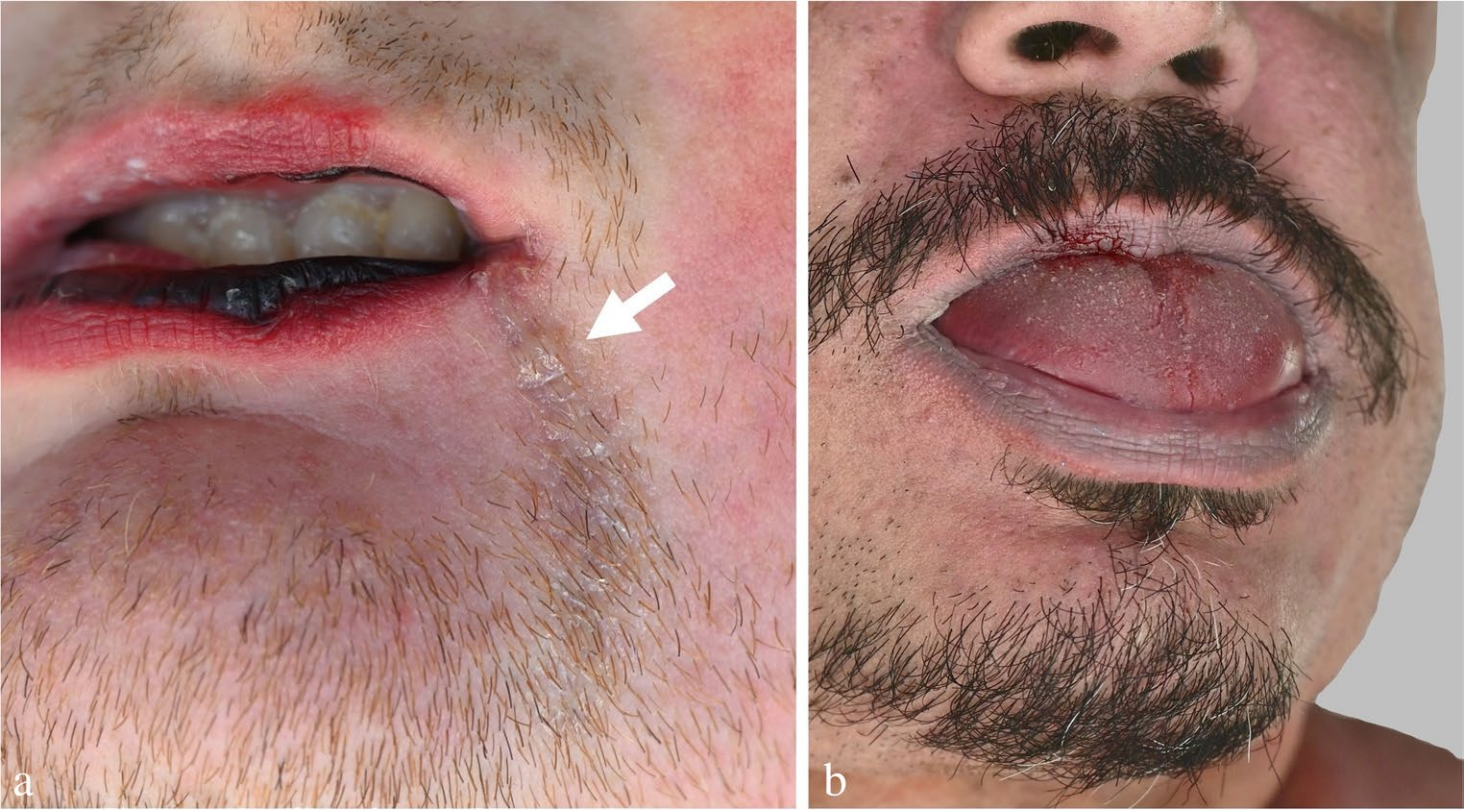
(**a**) Dried trickle pattern from saliva dripping from the mouth of a hanging victim (arrow). (**b**) Protrusion of the tongue in a victim of suicidal hanging

One important, yet often neglected aspect is the detection of biological traces on hanging devices and interposed textiles. The upper epidermal layers of the neck skin are often abraded by contact with the constricting noose and thereby transferred to the ligature (Fig. 1c), where they can be detected visually and/or by using molecular biological methods [44]. Beside epidermic particles, serous and fatty tissue fluids may be deposited on the loop, especially adjacent to squeezed skin folds. On the other hand, material from the hanging device occasionally sticks to the neck skin and/or to the victim’s hands, preferentially under the edges of the nails. A method to sample adherent fibres has been described by Frei-Sulzer already in 1955 [45]. In cases of severe facial congestion, blood can escape from the nostrils and/or ears causing respective trickle patterns (Fig. 2a, b). Analagously, other body fluids such as saliva, nasal discharge and sperm may leak from the respective orifices (Fig. 3a).

**Fig. 4:**
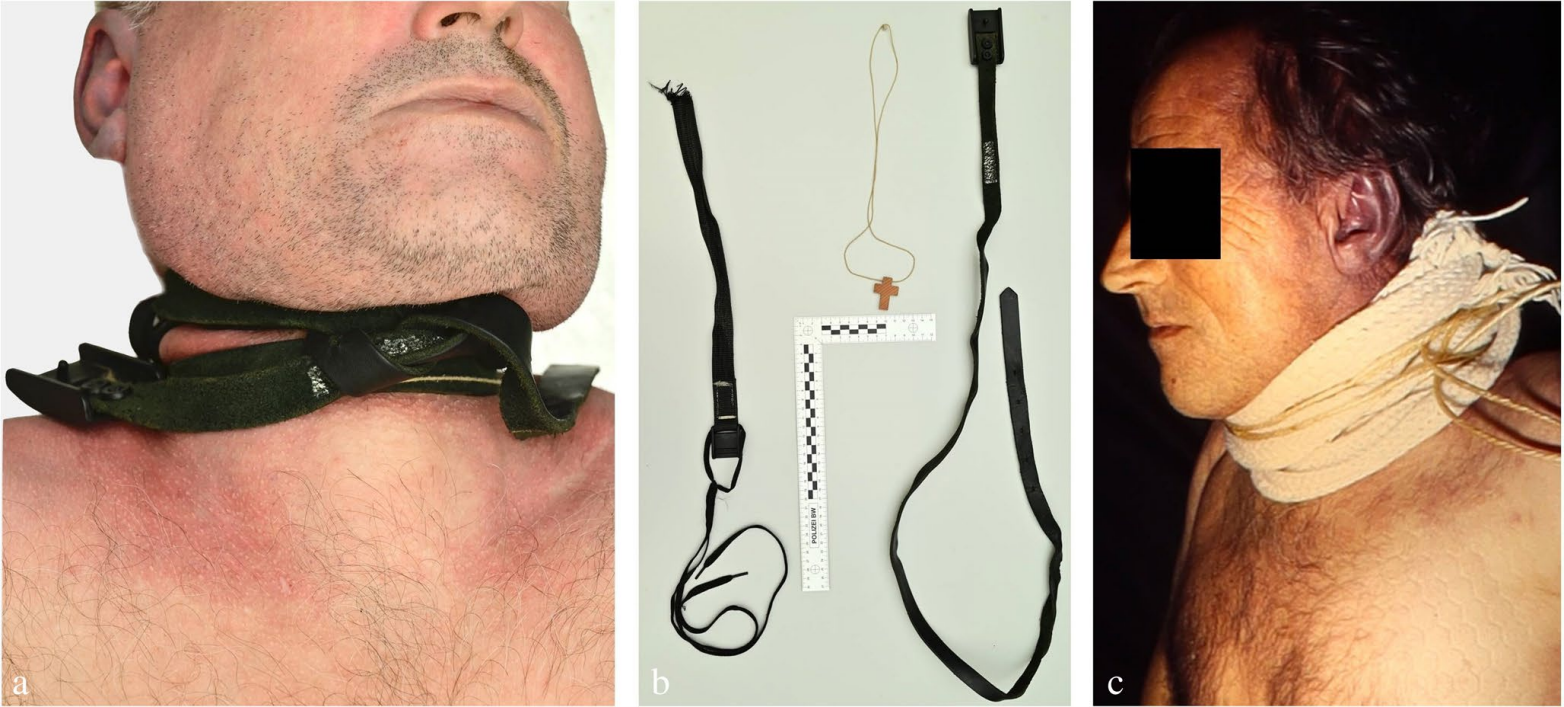
(**a**) Suicidal hanging by using two plastic straps. (**b**) A necklace with a cross-shaped pendant was interposed between the ligature turns. (**c**) Suicidal hanging with padding of the noose by an interposed scarf (cf [23])

An essential prerequisite for the holistic assessment of a hanging case is taking into account the victim’s clothing. One example of this are autoerotic hangings which may be characterized by either a naked body (Fig. 1a, b) or special dresses pointing to a sexual background (often associated with cushioning of the noose, self-tying, bondage, the presence of fetishes, penile strangulation and pornographic material) [23, 46–49]. Self-tying, gagging, sealing of the respiratory orifices by adhesive plaster and protective padding of the ligature are sometimes seen in cases of sexual asphyxia, but they also occur in suicidal hangings (Fig. 4c) [50]. In persons who climb a tree to attach the hanging device, hands and shoes may be discoloured from the bark [51].

### Gross external appearance

In hanging deaths, the body posture is decisive for the temporal formation, distribution and local intensity of *hypostasis*. If there is no contact between the suspended body and the floor or any other support, the post-mortem lividity will be located circumferentially on the limbs hanging down, especially the lower arms and hands as well as the legs and feet including the plantar regions (cf. Figure 1a, b). This distribution pattern of post-mortem lividity has been compared with gloves and stockings, respectively. Skin areas which are exposed to local pressure (such as from wristbands, garters and elastic bands) may be exempted from the discolouration.

**Fig. 5:**
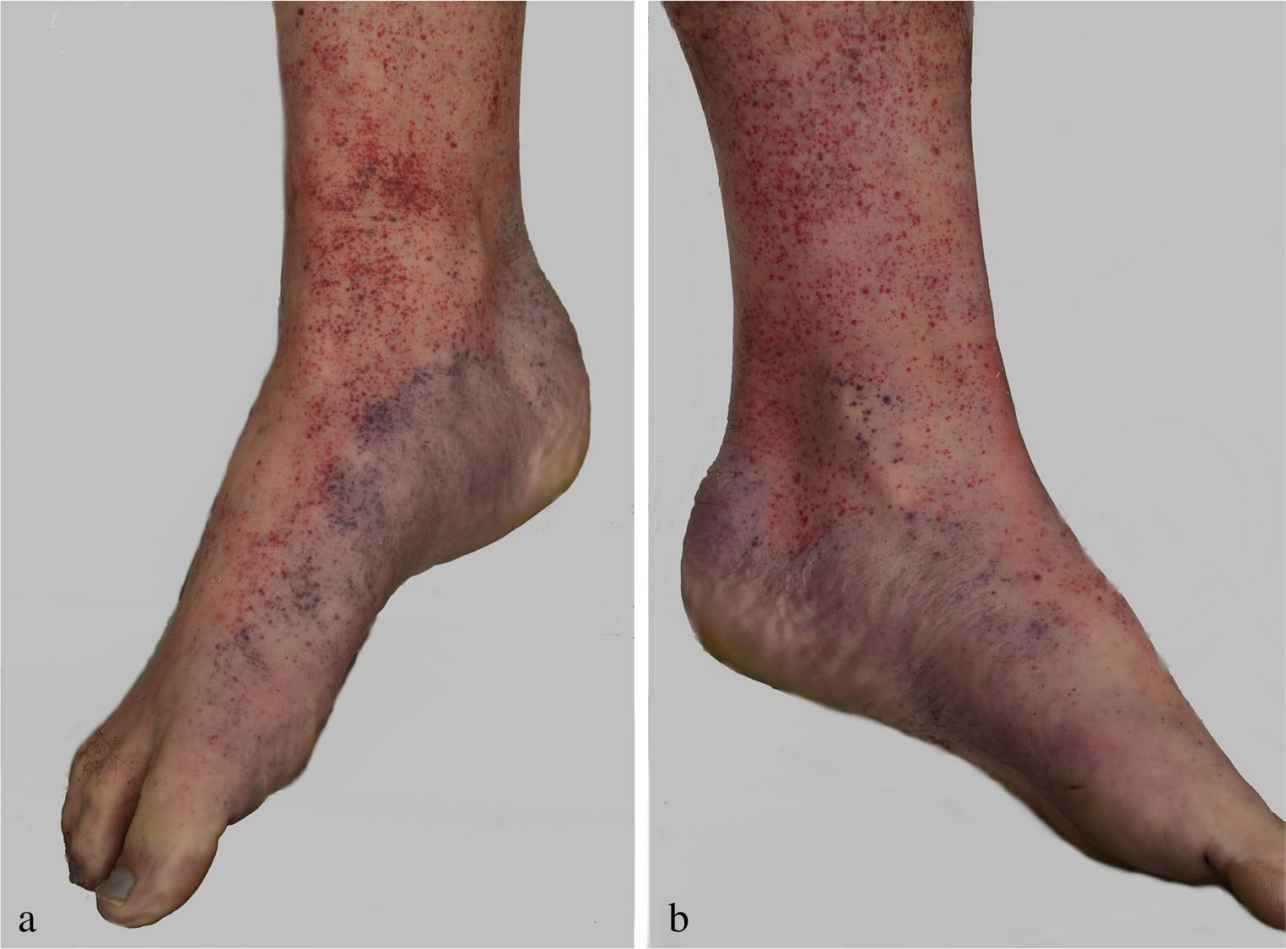
Intracutaneous blood extravasations in the right (**a**) and left (**b**) foot due to hypostasis in a case of hanging

As is known from other hypostatic body regions, the skin is often spotted with numerous intracutaneous haemorrhages which of course do not constitute proof of vital hanging (Fig. 5). Apart from the limbs, post-mortem lividity is typically found in the chin region, if the noose ascends to the back of the head, as well as on the skin areas above the hanging mark and the belt line, but also on the lower abdomen and in the genital region. If the victim is rescued from the hanging position at an early stage, the distribution pattern of hypostasis will change according to the secondary body posture.

When *rigor mortis* arises, the limb conformation gets fixed. So, a foot placed on a ladder rung or another support may cause a bent position of the ipsilateral hip and knee joint (cf. Figure 1a, b). Similarly, a stiffened elbow flexion can be due to a hand’s interposition between noose and neck [43].

The body parts, which are blood-rich due to hypostasis, tend to decompose quickly (skin discolouration, marbling, detachment of the epidermis, blistering). On the other hand, anaemic skin areas are prone to dehydration with subsequent mummification, especially in circulating and dry air. In cases of a prolonged period between hanging and discovery of the body, maggot infestation and decomposition processes may favour a dehiscence of the craniocervical junction finally resulting in decapitation [51].

## Hanging mark

Without doubt, the hanging mark is the most specific sign indicating a previous constriction of the neck by a noose subjected to the gravitational force of the suspended body. Its external appearance may already provide information about the shape characteristics, width and position of the ligature [52]. There are two main mechanisms causing a hanging mark: (1) the friction exerted by the tightening noose, often accompanied by shearing off epidermal scales which may be transferred to the surface of the hanging device (cf. Figure 1c); (2) the surface pressure of the loop affecting the neck skin. Continuous compression is followed by local displacement of the tissue fluid and results in drying, hardening and potentially a trough-shaped deepening of the hanging mark, preferentially on the opposite side of the knot and in cases of a prolonged suspension period.

**Fig. 6:**
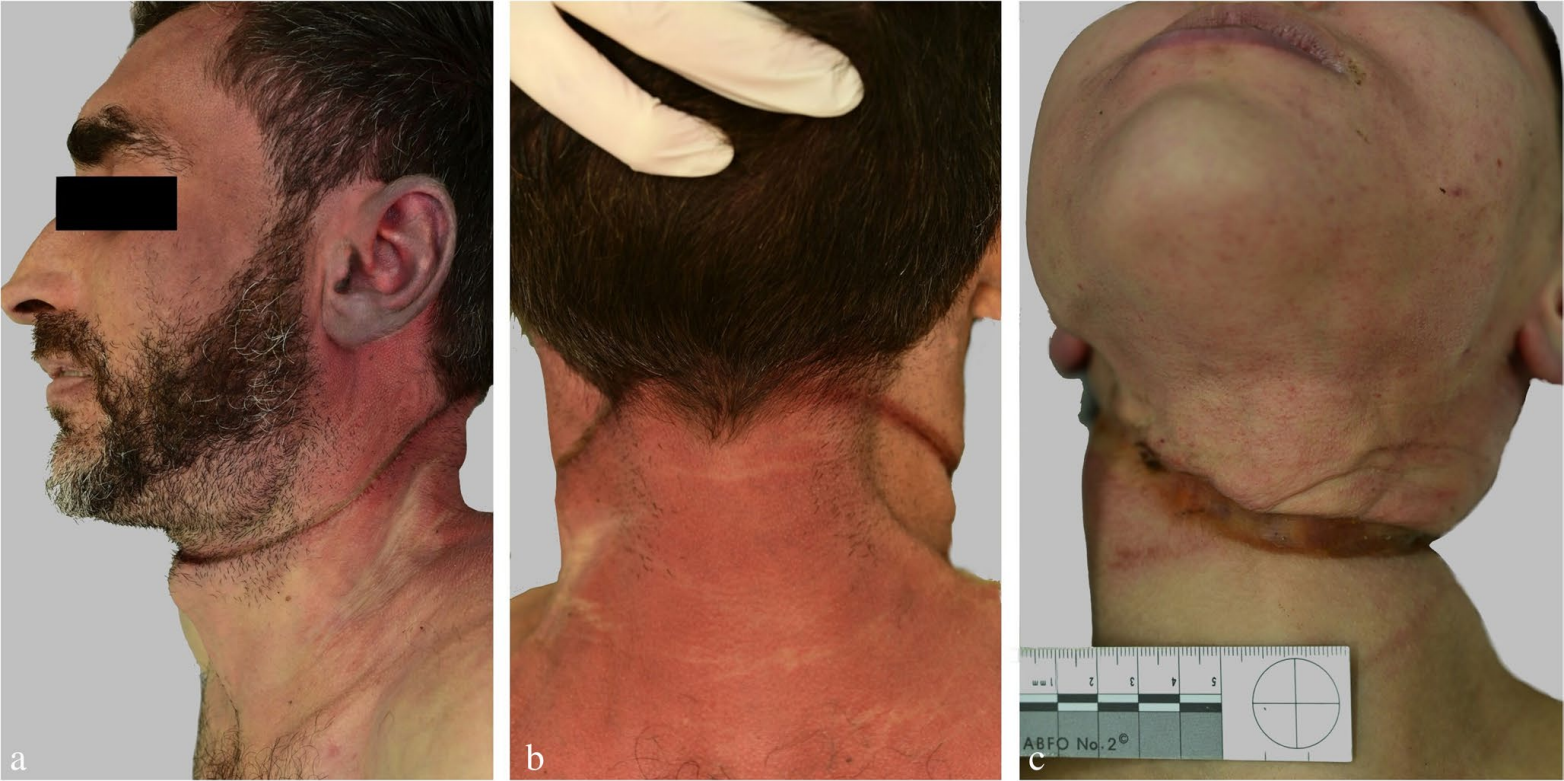
(**a**, **b**): Hanging by dorsal suspension with the ligature mark rising to its highest point in the transition area between the nape and the occiput. In the anterior neck region, the hanging mark crosses the midline above the laryngeal prominence. (**c**) Lateral suspension with the knot being positioned on the right side of the neck

Compared to marks seen in ligature strangulation, most hanging marks are characterized by an uneven depth of the groove and its course ascending to the highest point of the noose (Fig. 6a, b). Nevertheless, these rules of thumb must not be applied in an uncritical manner: For example, in victims suspended with a slipknot, the hanging mark may be evenly pronounced encircling the neck almost horizontally [53].

**Fig. 7:**
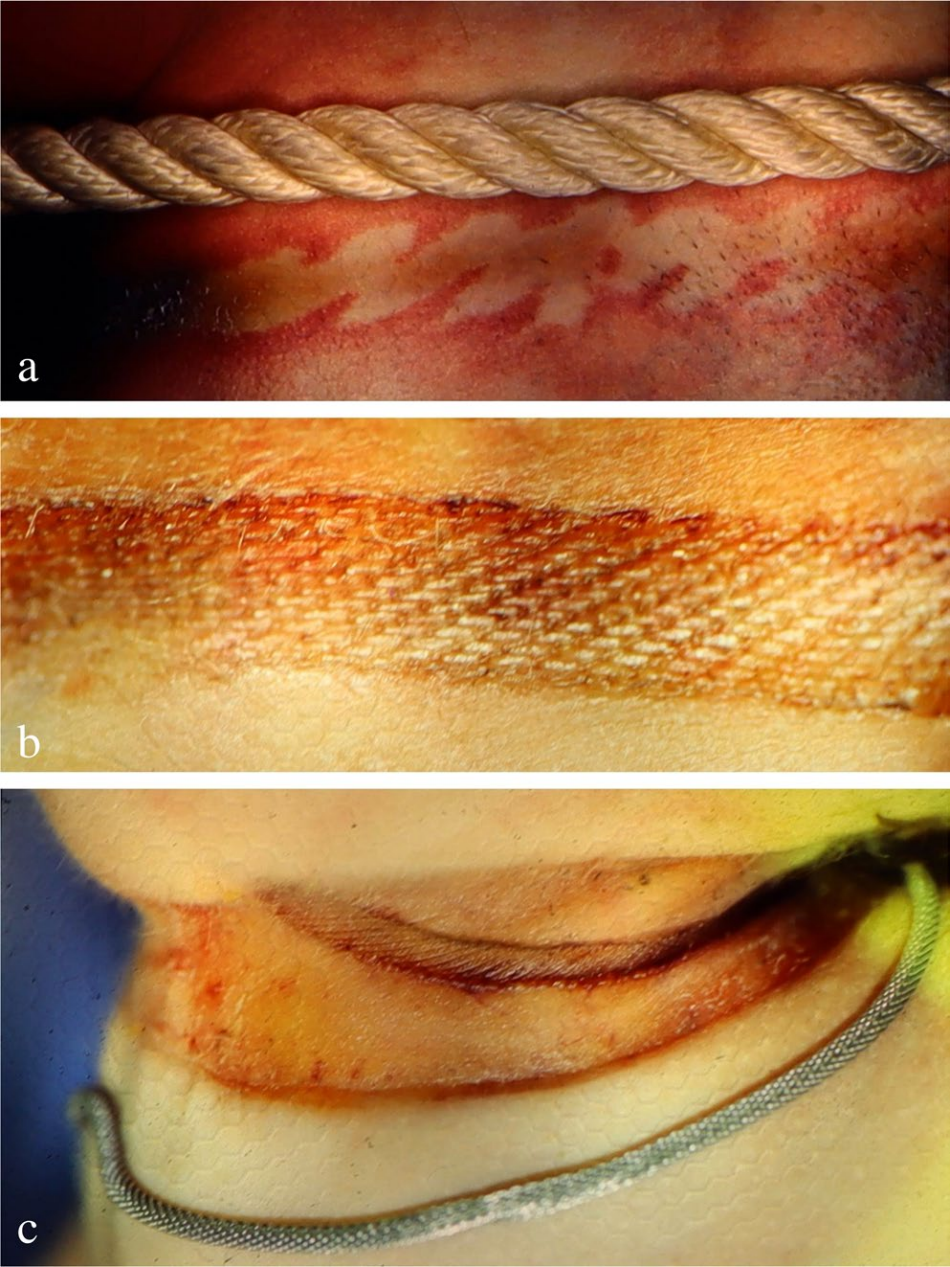
Patterned hanging marks caused by devices with a relief-like surface. (**a**) Pale skin areas corresponding to the elevated parts of a twisted rope with the margins being reddened by local hyperaemia (cf [23]). (**b**) Imprint on the skin reflecting the woven structure of the loop material. (**c**) Imprint of a necklace interposed between the hanging noose and the underlying neck skin

The *external appearance* of a hanging mark is mainly dependent on three influencing factors: (1) the drag exerted by the body weight or part of it; (2) the duration of suspension, (3) the width, texture and material properties of the hanging device. In extreme cases, there may be no distinct hanging mark at all (e.g. if a broad noose made of soft material was acting on the neck for only a short time) [24, 54]. On the other hand, the ligature can leave an imprinted pattern of its twist or weaving structure (Fig. 7). If there is no abrasion, some hanging marks present as a strip-shaped gap of the post-mortem lividity.

Up to the late 19th century, some authors argued that vital hanging marks are accompanied by congruent blood extravasations in the subcutis. Nowadays it is agreed that this is only true for (primarily) survived hanging attempts and in victims who underwent resuscitation procedures [23, 55]. Under such circumstances, the hanging mark may be level with the surrounding skin or even protrude from it (in the presence of tissue oedema and/or concomitant haematoma) [56].

**Fig. 8:**
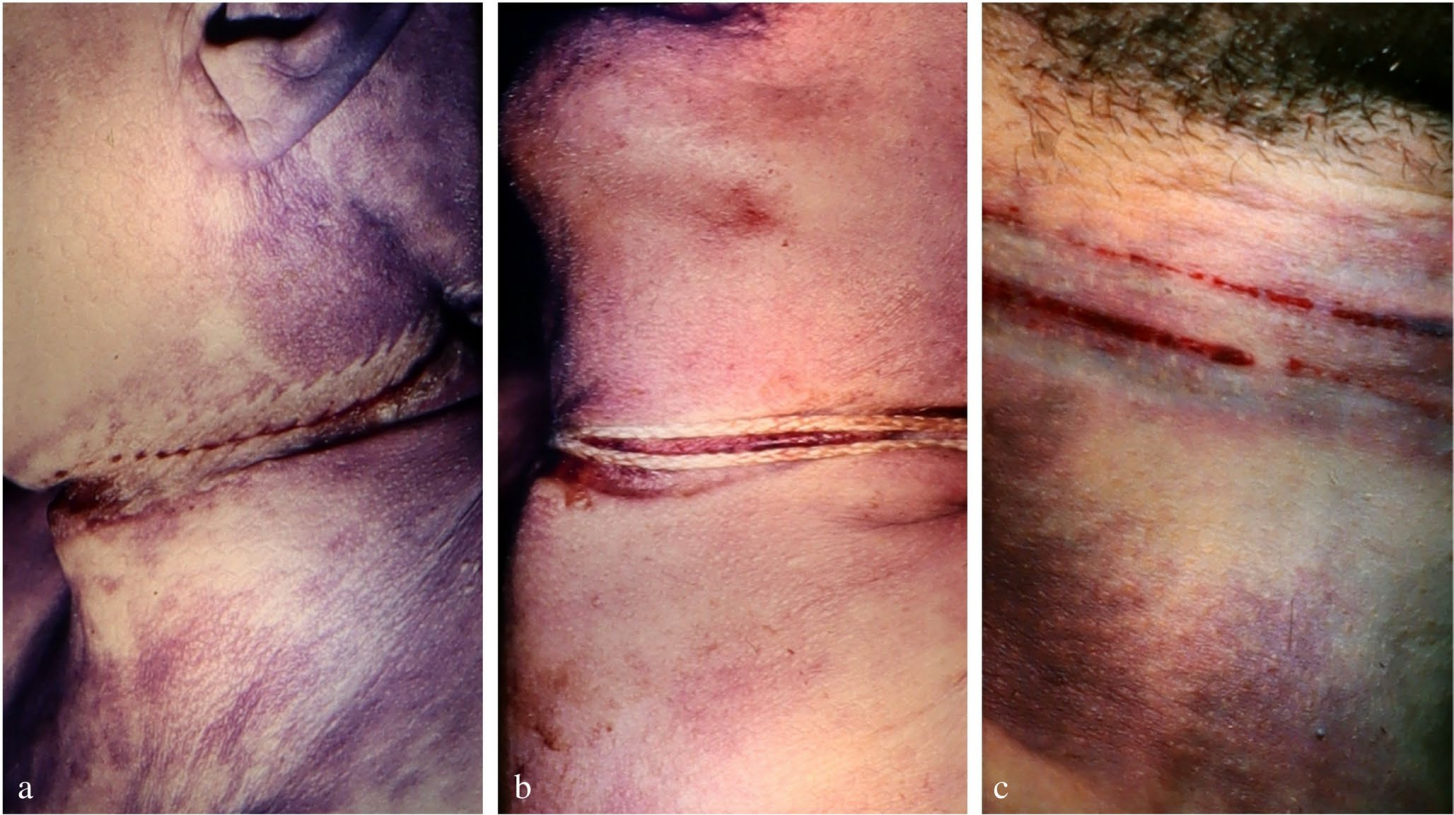
Intracutaneous haemorrhages on skin ridges between ligature turns running close together (cf [23])

*Ecchymotic skin* ridges (Fig. 8), which are frequently found between the ligature turns in cases of dual or multiple loops, have erroneously been considered a vital sign until present times, though evidence to the contrary dates back to the late 19th century [28]. Several authors have shown that extravasations between ligature turns can be produced many hours after death, even outside hypostatic skin areas. This also applies to *blisters* filled with serous fluid and fat on squeezed skin folds between ligature turns and adjacent to them [29, 30, 37, 44, 57, 58]. Extravasations and blistering in close proximity to dual/multiple ligature marks cannot prove ante-mortem hanging, as these findings may also be produced post-mortem. Regardless of a vital or post-mortem formation, the skin located immediately below the hanging furrow may show shallow tears due to overstretching [59].

In some constellations, the hanging mark is inconspicuous [24]. It is therefore not surprising that deaths by hanging can be overlooked by the medical examiner, if the body is no longer suspended and the noose has been removed [54]. On the other hand, spurious signs may mislead the forensic pathologist by suggesting a hanging mark: In a deep neck fold the formation of post-mortem lividity is prevented by local pressure so that a pale linear stripe appears when the skin is stretched [27]. After refrigeration of a body, skin indentation from a neck crease potentially imitates a furrow due to the congealing of the subcutaneous fat [27]. In prone position, contact with a hard object may cause brown and leathery skin discolourations similar to abrasions [60]. Post-mortem artefacts from ant bites can also mimic excoriations [27]. A chin strap attached by the undertaker to keep the deceased’s mouth closed might produce a furrow resembling a hanging mark. In dressed bodies, livor is often blanched under a tight shirt collar thus causing a pale skin mark. Similar artefacts are seen in patients treated with a cervical collar to support the neck or with ribbon-like fasteners of an oral endotracheal tube [27].

In some instances, the hanging device tightens step by step during suspension until the noose reaches its final position. The successive upward movement of the ligature may produce a widened or a second mark [61].

## External findings above the level of neck compression

*Tongue protrusion* is a common finding in hanging fatalities (cf. Figure 3b). According to a retrospective study from Thailand, tongue protrusion was found in 83/204 cases of incomplete (partial) hanging and in 29/40 cases of complete hanging [62]. The exposed tip of the tongue is mostly dark coloured due to drying, but not bruised. The displacement of the tongue is due to the suspension-induced shift of the upper neck structures including the mouth base and does not represent a sign of vitality [63]. The same applies to salivation producing vertical tracks in the chin region and on the clothing below (cf. Figure 3a) [29, 63].

**Fig. 9:**
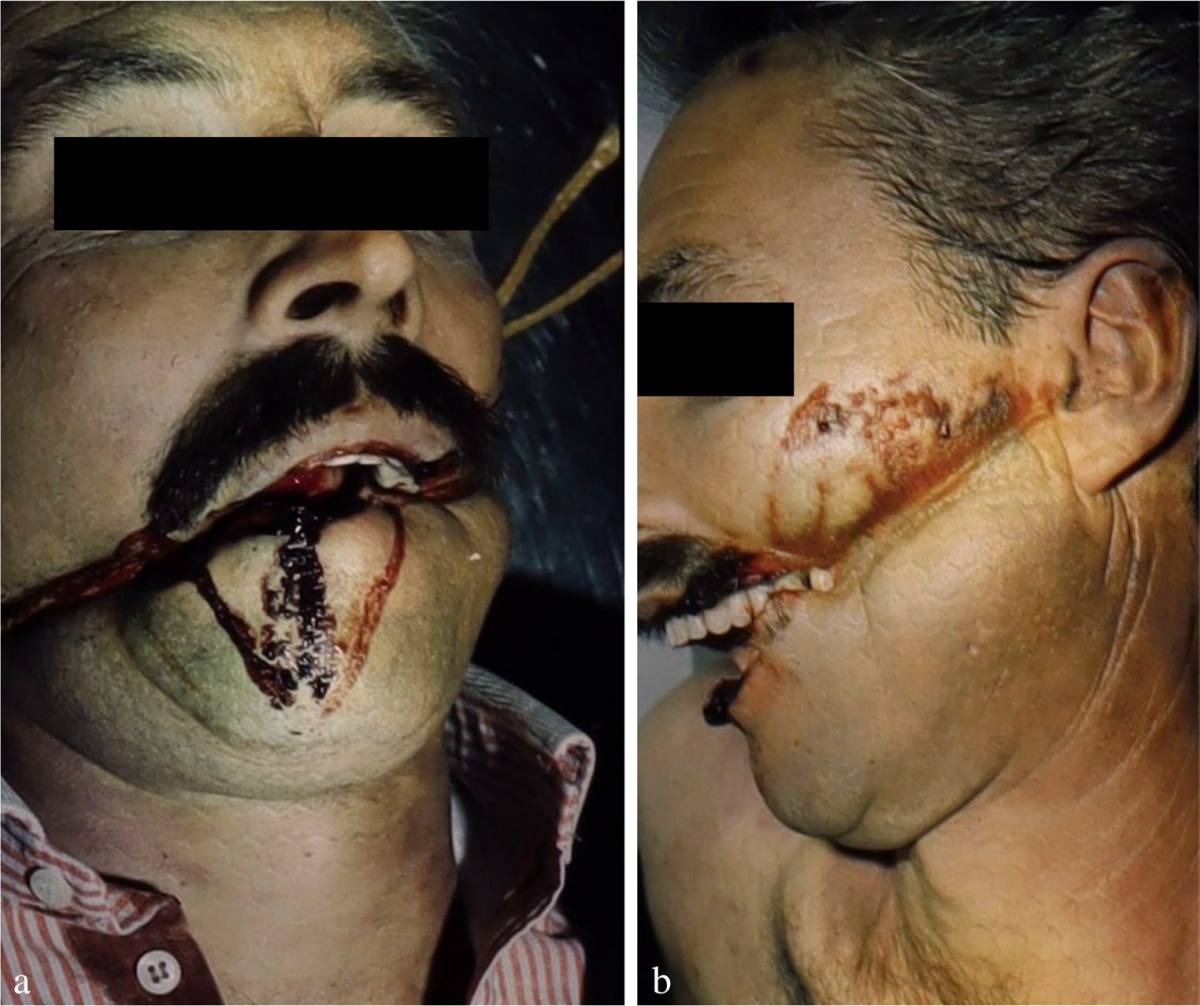
Supracervical hanging with the noose running through the mouth (cf [64])

Bluish skin discolouration away from the hypostatic body regions is a frequent finding in areas above the ligature, mainly in cases of incomplete suspension and in ventral or lateral knot positions (cf. Figure 6c) [36]. The causative mechanism of supracervical blood accumulation is compression of the neck veins draining the blood from the head to the thorax, while the arteries are still patent (at least partially). *Venous congestion* above the ligature mainly occurs in bodies being supported to a great extent [32, 34, 36, 59] such as in lying, kneeling and sitting positions. The tensile forces necessary to occlude the venous and arterial neck vessels have been determined by Schwarzacher [32]. Even in supracervical hangings, where the noose runs above the neck (e.g. through the mouth), the pressure exerted on the carotids and vertebral arteries can cause death due to brain ischaemia (Fig. 9) [34, 64].

**Fig. 10:**
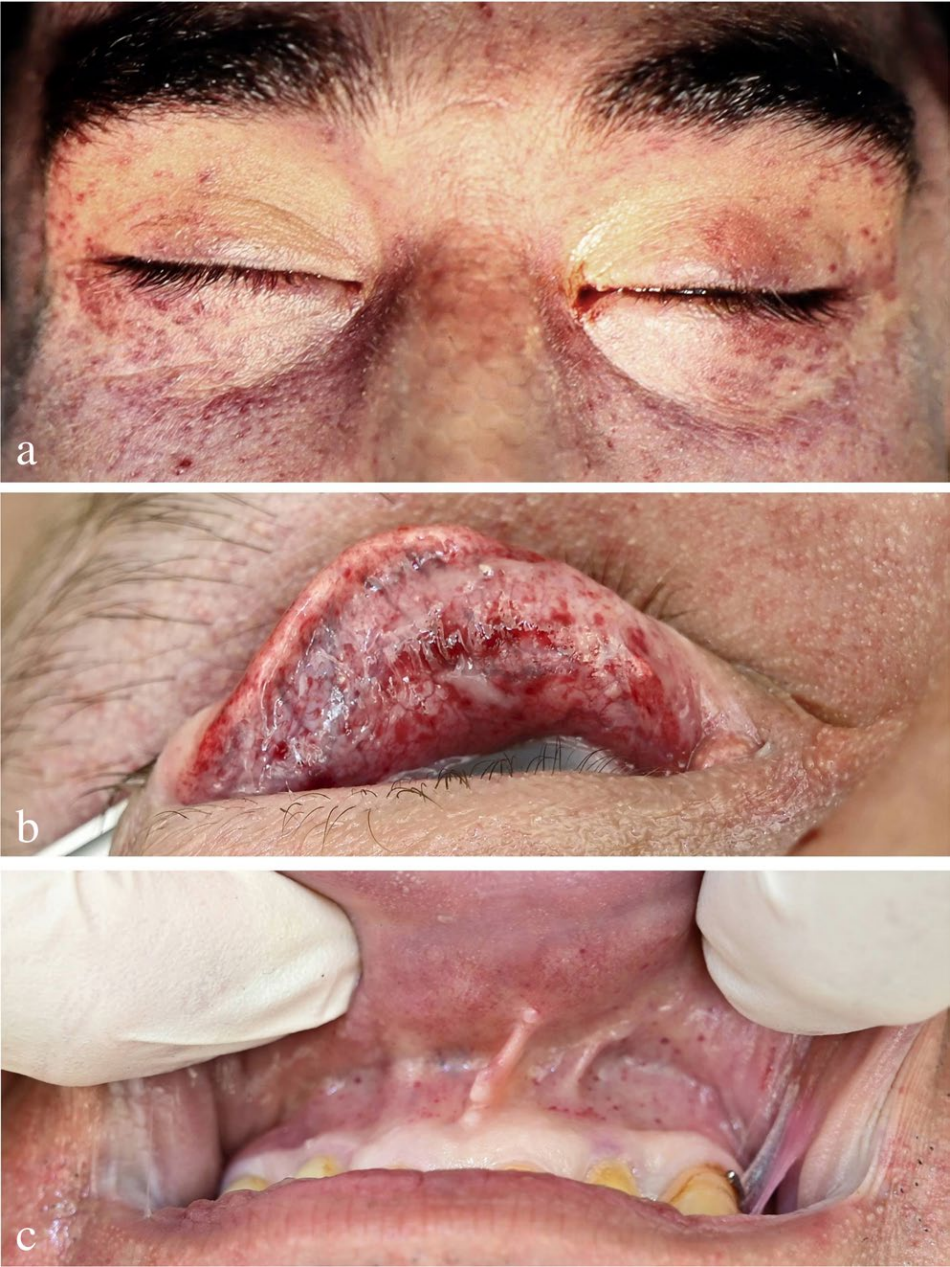
Suicidal hangings with signs of venous congestion above the level of strangulation. (**a**) Punctiform skin bleedings in the eyelids. (**b**) Petechial bleedings in the conjunctiva of the upper lid. (**c**) Mucosal petechiae in the oral vestibule

The emergence of *petechial bleedings* in the conjunctivae, the oral mucosa and the facial skin (Fig. 10) depends on different factors such as the course of the noose, the position of the knot, the extent of any support and the time period of neck compression. Thus, it is hardly surprising that the reported frequency of petechiae in hanging deaths varies over a wide range. According to Reuter, the overall percentage may be in the order of 20–30% [33]. Higher shares up to 72.7% [65–67] can be accounted for by the fact that highly atypical hangings are overrepresented in the forensic autopsy material. Geserick and Kämpfe described the percentage of conjunctival bleedings in the range of 48% [68]. Especially in cases of prolonged survival or initially successful resuscitation attempts, the conjunctival petechiae often merge forming a continuous haemorrhage (hyposphagma) [55]. In freely suspended hanging victims, the presence of petechiae may evoke suspicion of prior strangulation by manual or ligature strangulation [69].

It was rightly stated that petechial haemorrhages above the level of strangulation are not caused by asphyxia (i.e. as a result of oxygen deficiency), but as a consequence of vessel ruptures due to venous blood congestion [69]. If other potential causes can be excluded, petechial haemorrhages may be considered as a sign of vital suspension indicating a pressure-related impairment of the venous drainage from the head to the thorax.

## Internal findings

In order to avoid *artefacts* from post-mortem blood extravasation, removal of the heart and brain prior to dissection of the neck is recommended [27, 53, 61]. This technique drains the neck veins of blood and thus reduces the risk of preparation-related vessel injury with consecutive bleeding [70]. Prinsloo and Gordon [71] described artefactual post-mortem haemorrhages between the oesophagus and cervical spine.

**Fig. 11:**
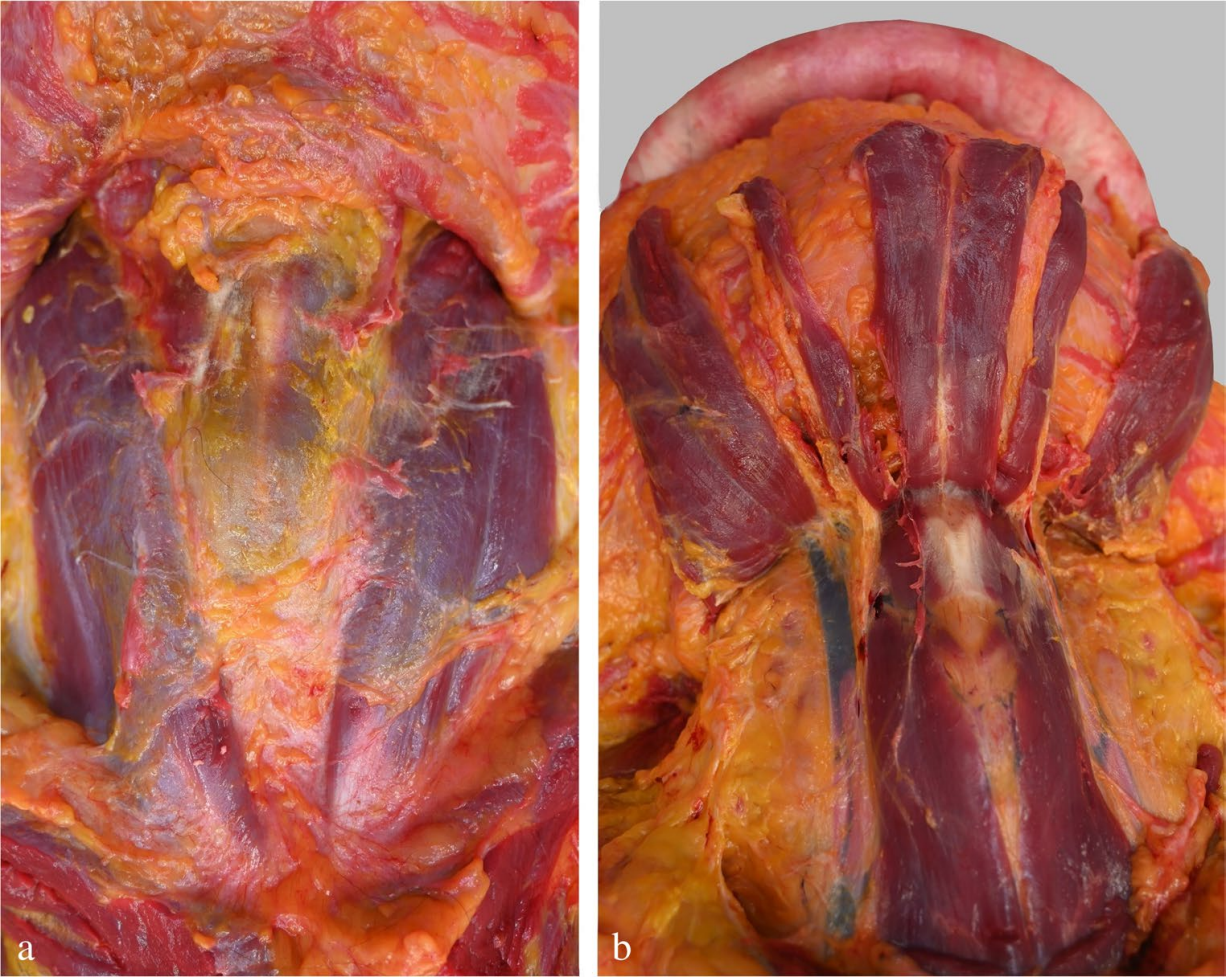
Layer-wise dissection of the anterior neck region in a victim of suicidal hanging. (**a**) The subcutis is free of haematomas. (**b**) The neck muscles are individually represented and do not show any blood extravasations

**Fig. 12:**
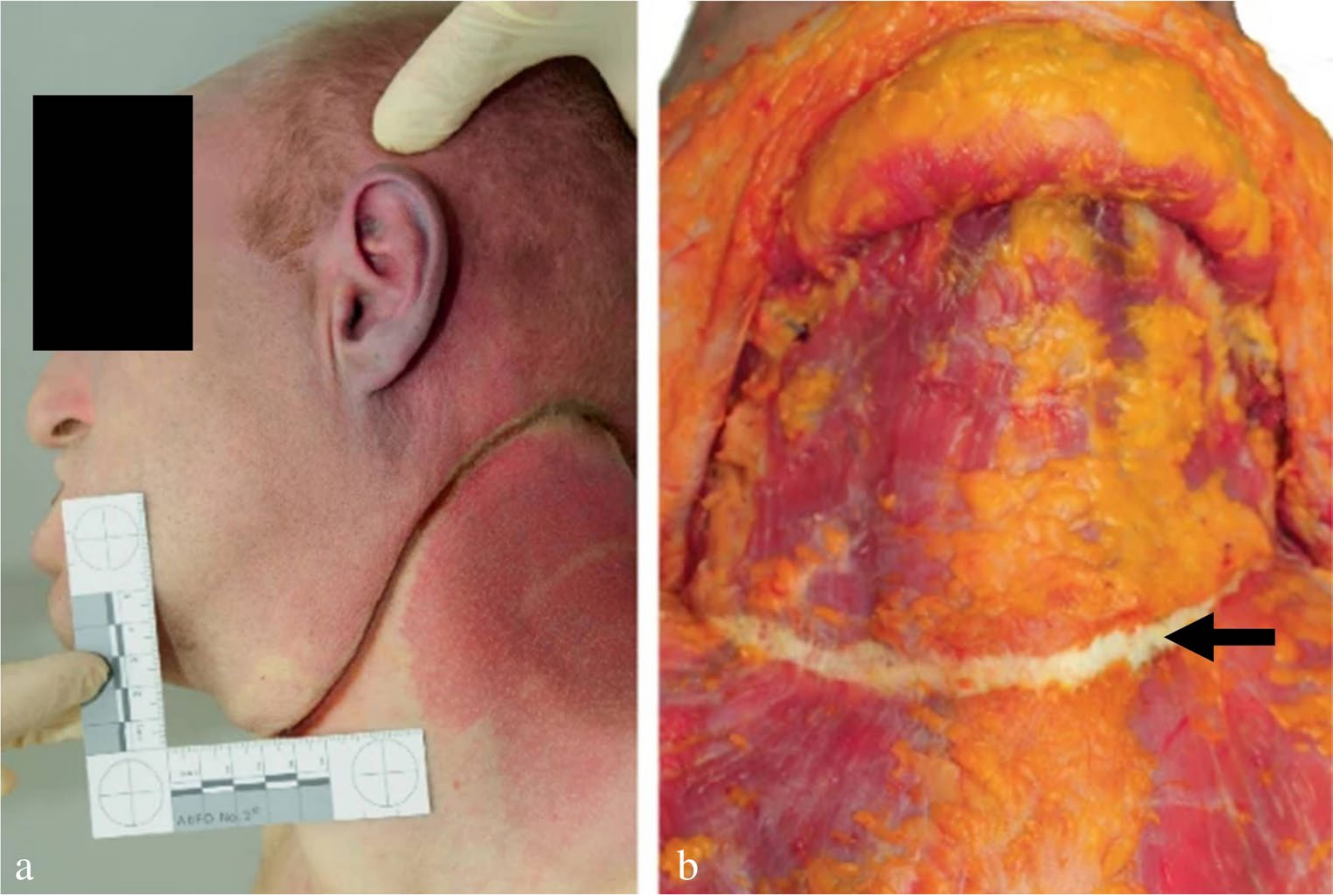
External (**a**) and internal (**b**) hanging mark in a case with dorsal suspension. The hanging device was a shoelace. Due to the prolonged pressure exerted by the loop, the underlying subcutis shows whitish discolouration (cf [51])

In cases of hanging, there is usually no subcutaneous haematoma under the *ligature mark*, as in primarily fatal acts such bleedings are prevented by persistent compression of the tissue (Fig. 11). Nowadays it is generally agreed that significant bleeding under the hanging mark is restricted to surviving victims [55] and those who underwent attempts at resuscitation. Nevertheless, on histological examination a hanging furrow may show minor haemorrhagic infiltration of the deep dermis at the transition to the subcutis [72]. In some cases, especially those with a prolonged suspension time, a so-called *‘inner hanging mark’* is visible to the naked eye. It is characterized by a whitish-grey discolouration and reduced thickness of the subcutaneous layer under the dried-out skin furrow (Fig. 12).

For many decades, studies on hanging marks have been performed to distinguish ante-mortem and post-mortem infliction. Metachromasia in dermal collagen has turned out to be an unreliable indicator of vitality [73]. The same applies to the esterase activity of the affected skin and its histamine content [69]. In the recent past, several authors focused on *immunohistochemical reactions* possibly indicating the vitality of a hanging mark. For detailed information reference is made to the relevant special literature [12, 13, 74–78]. According to Kondo [73], the aquaporin-3 expression in the keratinocytes is enhanced in ligature marks.

**Fig. 13:**
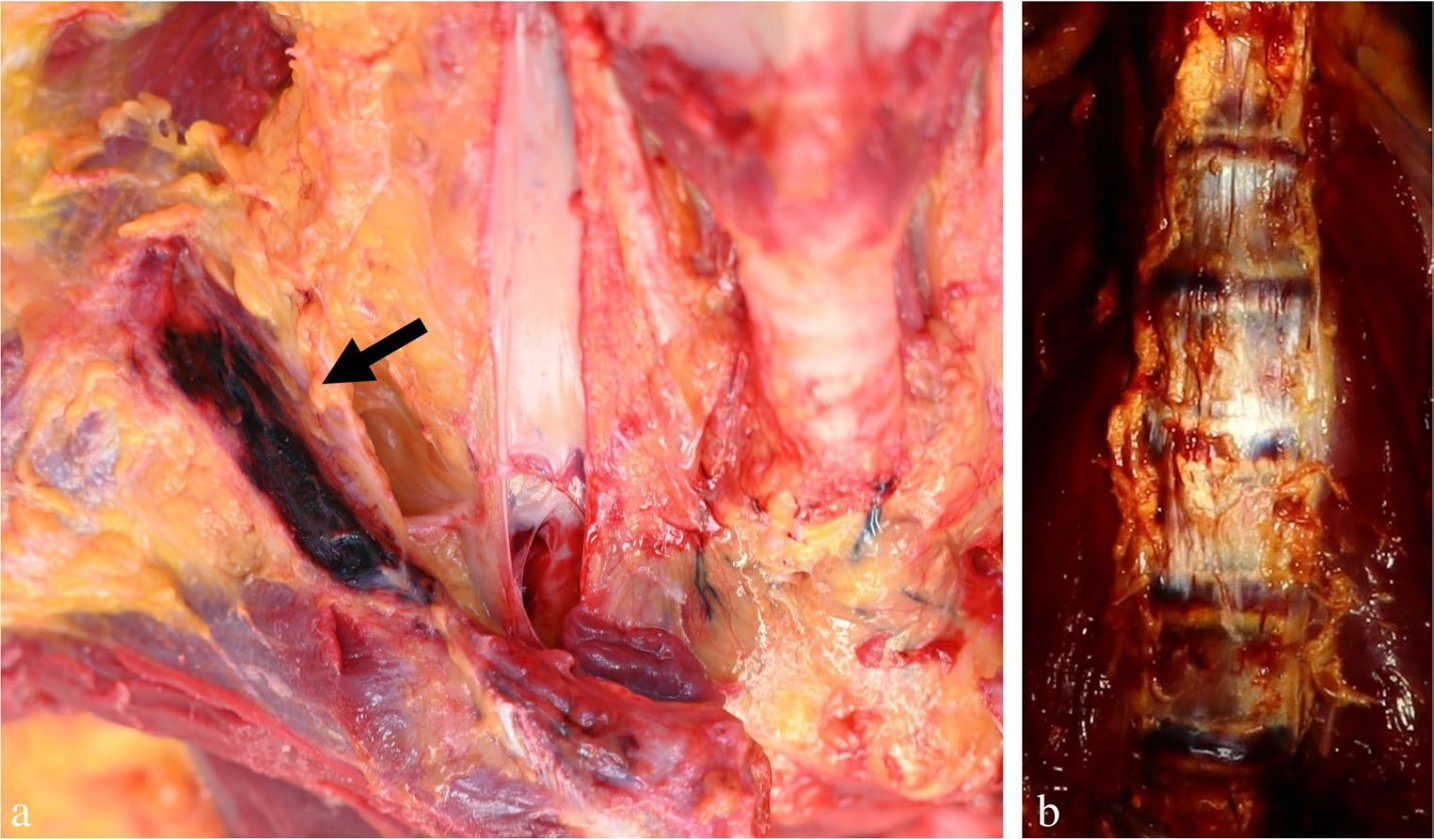
(**a**) Anterior neck region of a hanging victim after layer-wise dissection including removal of the right sternocleidomastoid muscle, whose origin is infiltrated with blood (arrow). (**b**) Frontal view of the lumbar spine showing Simon’s haemorrhages corresponding to the intervertebral disks (cf [23])

Among the cervical *muscle haemorrhages* observed in hanging deaths, the ones located at the clavicular origin of the sternocleidomastoid and – to a lesser degree – at the sternal origin are the most important (Fig. 13a). The blood extravasations are not confined to the muscle origin, as they also extend to the adjacent periosteum. Traction forces deriving from the suspended body with consecutive stretching of the exposed neck muscles are considered to be the main causative mechanism. According to Keil et al. [79], the bleeding occurs more often on the side of the highest point of the loop. A recent study [80] indicated that the greatest frequency was found in typical hangings with complete suspension. Attempts at resuscitation were not supposed to promote the incidence of periostal blood extravasations. In victims of manual and ligature strangulation, haemorrhages are typically localized within the muscle tissue and not in the periost of the clavicular origin.

Similar to Lim et al. [80], Hejna and Zátopková [81] observed an association between bleedings at the muscle origins and a more or less complete suspension of the body. Furthermore, the authors could confirm that there is a statistical correlation between the position of the knot and the frequency of haemorrhages. Comparable results were obtained by Leković and Nikolić [82]. The review of Blandino et al. [21] lists numerous case series published so far with striking differences in the reported incidence of neck muscle haemorrhages ranging from 6 to 96.3%.

Though the presence of blood extravasations seems to suggest hanging during lifetime, already in 1979 Kerde and Heuschkel [29] stressed the possibility of post-mortem causation. In the respective article, the authors arrived at the following conclusion: Among all the macroscopic signs of hanging described so far, only facial petechiae as seen in cases of atypical hanging and Simon’s bleedings could not be produced post-mortem. So, the mere existence of neck muscle bleedings should be interpreted with caution.

Layer-wise dissection of the cervical soft tissues (after removal of the heart and brain) increases the probability that even minor blood extravasations can be identified at autopsy [83–85]. From the perspective of the injuring mechanism, haemorrhages at the origins of the sternocleidomastoid muscles are neither pathognomonic for hanging [81] nor for a certain manner of death.

**Fig. 14:**
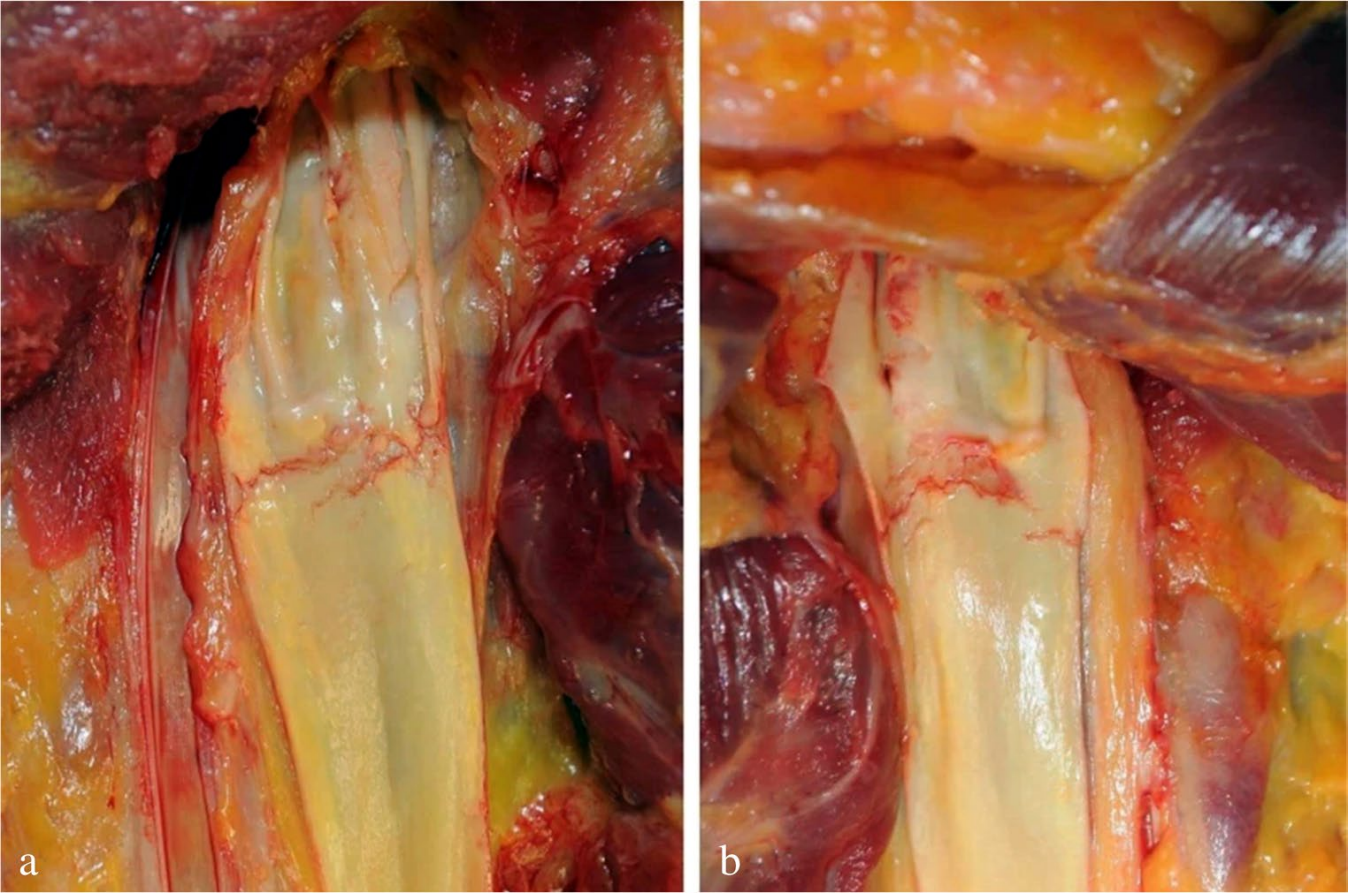
Amussat’s sign: Transverse tension cracks of the intimal layer located in the right (**a**) and left (**b**) common carotid artery just below the bifurcation (suicide by hanging in a 43-year-old man, cf [85])

A well-known finding in the context of hanging is the so-called *Amussat’s sign*. The term refers to intimal tears of the common carotid arteries, mostly located just below the bifurcation. The lesions are typically multiple, cross to the vessel’s longitudinal axis and in a parallel arrangement (Fig. 14). The intimal tears are considered to be caused by a combination of direct compression and indirect stretching due to the gravitational drag of the suspended body [86]. Amussat’s sign is not specific for hanging, as it can occur also in cases of direct blunt traumatization and overstretching of the neck [86].

Literature data on the frequency of carotid lesions vary in a wide range up to 52.3% (see the compilation in [21]). The percentage reported by Hejna (16%) calculated on the basis of a prospective study [86] seems realistic. The results published by Laiho et al. in 1968 [87] were almost identical. According to Hejna [86], the incidence of Amussat’s sign increases with the victim’s age, but is independent of other parameters such as sex, weight, completeness of suspension and position of the ligature. In contrast, Nikolić et al. [88] found that Amussat’s sign was ipsilateral to the suspension when the knot was positioned laterally. In the retrospective study of Kurtulus et al. [89], the frequency of Amussat’s sign amounted to only 3.9%, and in all positive cases the hanging was classified as ‘atypical’. Samarasekera and Cooke [90] observed intimal tears of the carotids more often in complete hanging compared to partial suspension. Blandino et al. [21] rightly pointed out that the currently available data on Amussat’s sign do not allow valid conclusions to be drawn for statistical associations with the type of hanging. Luigi Crudele et al. [91] recommended immunohistochemical staining of carotid intimal lesions for glycophorin A aiming at a better identification of concomitant blood infiltration. With regard to the vascular lesions accompanied by local bleedings, their (limited) relevance as signs of vitality should be assessed with caution as all the other haemorrhages in the neck region.

Apart from intimal tears of the carotid arteries, several other vascular lesions have been documented in connection with hanging. Martin’s sign is defined as a haematoma in the adventitia of the carotid artery [21, 86, 92]; it may be associated with Amussat’s sign. Asirdizer and Kartal [11] as well as Blandino et al. [21] reviewed papers about further vessel-related findings such as subintimal bleedings of the carotid artery, tears in the internal or external carotid artery and transverse lesions of the jugular veins. Recently, Lange-Herr et al. [93] and Kovařik et al. [94] reported on novel aortic lesions in hanging deaths, namely haemorrhages at the junctions of the posterior intercostal arteries, aortic intimal breaching as well as subintimal and periadventitial haemorrhages.

In victims of death by hanging, the *tongue* may show two kinds of intramuscular haemorrhages: (1) bruises in the anterior and lateral parts due to compression between the dental arches (especially in cases of at least temporary survival or after attempts at resuscitation); (2) blood extravasations in the central parts and in the root of the tongue due to severe venous congestion (particularly in cases of incomplete and/or atypical hanging; cf. Figure 2c). If a protruded tongue remains clamped between the dental arches until cardiac arrest, the squeezed areas usually don’t show any bruising [95]. This is attributable to the local pressure exerted by the clenched teeth preventing blood extravasation. Massive tongue bleeding from venous congestion is usually attributable to ‘abnormal‘ position of the noose [96].

In the past, post-mortem findings such as blood fluidity have been considered as a sign pointing to asphyxia. The more recent literature emphasizes the lack of specificity so that a liquid state of the cadaveric blood cannot be regarded as indicative of asphyxial death.

## Hanging-related injuries of the hyoid and larynx

**Fig. 15:**
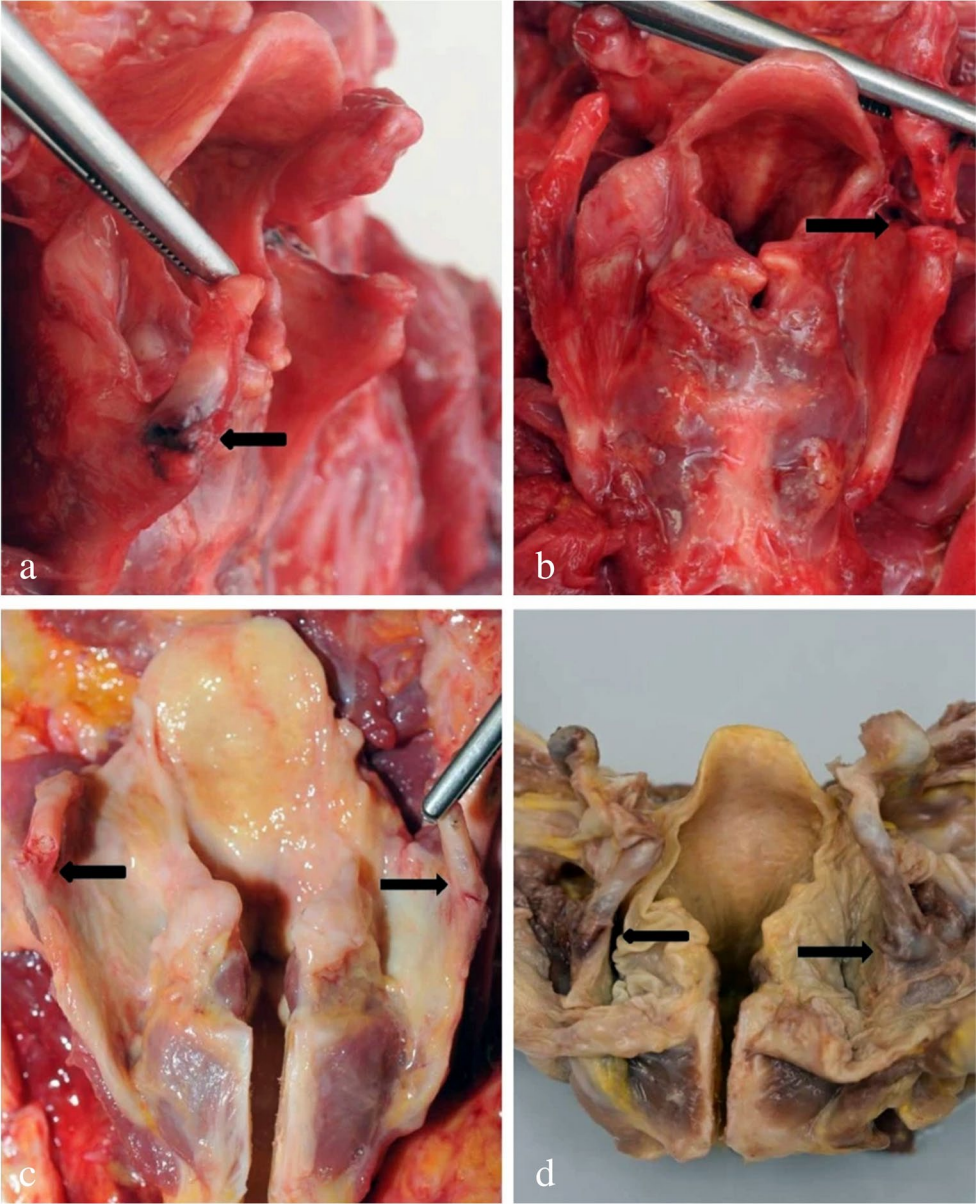
Injuries of the thyroid cartilage in three cases of hanging: fractures of the left and right greater horn (**a**, **b**) and of both greater horns (**c**). (**d**) Anatomical preparation of the larynx (fixed in formalin) showing post-mortem fractures of the greater horns (arrows) caused by improper handling of the body during transport (no hanging death! )

Injuries to the *hyoid-laryngeal complex* were the subject of abundant studies which have been reviewed in several overview articles to which reference is made here [6, 8, 16, 21, 97–100]. Representative of numerous case series, special mention should be made here of Missliwetz [101] who evaluated 500 suicidal hangings: 34% of the victims revealed fractures of the thyroid cartilage, 20% fractures of both the thyroid cartilage and the hyoid and 14% isolated fractures of the hyoid. Comparable percentages have been reported by Laiho et al. [87] as well as Betz and Eisenmenger [102]. Typically, the superior horns of the thyroid cartilage and/or the greater horns of the hyoid bone are affected (Fig. 15). Fractures of the cricoid are rare in hanging deaths (with a frequency of only 2.6% in the autopsy material of Missliwetz [101]); they have been regarded as a potential pointer to homicide [103]. In this context, Reuter [33] stated: A fracture of the cricoid cartilage will always raise the suspicion of homicide simulating self-hanging. If a cricoid fracture is actually caused by a hanging noose, this may be attributable to an exceptionally low position of the ligature.

From the perspective of biomechanics, damage to the hyoid-laryngeal complex results from the traction and compression forces exerted by the noose: The spine restricts the backwards displacement so that the hyoid and the superior horns of the thyroid cartilage undergo critical deformation followed by fracture. External factors such as the victim’s weight, the drop height and the position of the knot have a major influence on the occurrence and location of any fractures. Among the internal factors, the victim’s sex and age are significant: In males and old people, the degree of ossification tends to be higher and accordingly the frequency of fractures is increased within these groups [8, 98, 99]. In contrast, victims with hyoids and laryngeal cartilages not yet ossified may lack structural damage even in cases of ‘typical‘ hanging.

Incorrect handling of the body during either transport or dissection can cause post-mortem fractures of the laryngeal horns [104]. Pollanen [105] described the main pitfalls encountered in the neck at post-mortem examination including variations in the developmental anatomy of the hyoid bone and triticeous cartilages located at the posterior end of the thyrohyoid ligament [84].

One might be inclined to interpret haemorrhage at the fracture site as an indicator of vitality [21]. The investigations of Kerde and Heuschkel [29], however, concluded that the mere presence of a fracture haematoma does not prove vital suspension. Therefore, a cautious assessment has to be recommended. Especially under difficult dissecting conditions (e.g. in putrefied or burned bodies), pre-autoptic imaging (X-ray examination, pmCT) is a highly beneficial tool for a non-destructive detection of any injuries [106–112]. In addition and complementary to the conventional preparation methods, stereomicroscopic investigation of the hyoid bone and laryngeal cartilages can provide valuable information [97].

Fractures of the hyoid-laryngeal complex are not necessarily caused by hanging or manual/ligature strangulation. They may also occur in other kinds of mechanical traumatization such as fall from height and traffic accident [113–115]. In forensic autopsies, a considerable number of the victims (5.6% according to Maxeiner [116]) reveal healed fractures; the affected persons are mostly chronic alcoholics prone to injuries from falls or violent assaults. Nontraumatic laryngeal fractures have been reported by Santamaria et al. [117]. In a case presented by White and Carver [118], an isolated hyoid bone fracture was attributed to self-induced vomiting.

Injuries of the *cervical spine* are mainly discussed in connection with long-drop hangings. Based on a retrospective autopsy study, Nikolić and Zivković [119] concluded that in suicidal short-drop hangings injuries of the cervical spine occurred in only 3.3% of the victims, most of them being at an advanced age. The majority of these lesions are restricted to non-osseous structures such as the anterior longitudinal ligament and the disks, usually without displacement of the vertebral bodies. Apart from (partial) ruptures of the ligament system and damage to the intervertebral disks, some other soft tissue injuries of the cervical spine have been reported including epidural haemorrhage as well as bleeding into the intervertebral foramina and the facet joints [120]. In contrast, the spinal cord is hardly ever affected. The presence of any blood extravasation originating from the above-mentioned lesions should be interpreted with caution, as the macroscopic appearance need not be different in fatalities with a short survival time and in neck injuries inflicted post-mortem [120].

In 2023, Glengarry et al. [121] published two cases of suicidal hanging associated with unilateral dislocation of the temporomandibular joint. The finding was identified on routine post-mortem computed tomography. In both instances, the dislocation occurred on the side opposite the point of ligature suspension.

*Cervical fractures* are mostly dealt with in the context of long-drop hangings. The abundant literature on this subject has recently been reviewed by Blandino et al. [21] and thus no amendment is needed. Special emphasis was placed on cervical fractures in victims of judicial hanging. In a survey of 34 relevant cases [122], the authors stated that the so-called hangman’s fracture of the axis vertebra is seen only in a small proportion of the victims. This type of cervical injury is characterized by a bilateral break of the pedicles [123]. The causative mechanism is hyperextension and distraction of the neck, as it is to be expected in long-drop hangings with the knot being placed under the mandible so that the head is jerked back when the noose tightens. A classical hangman’s fracture is, however, not restricted to dorsally flexed tractions and the pedicles cannot be regarded as the sole site of predilection [124, 125].

**Fig. 16:**
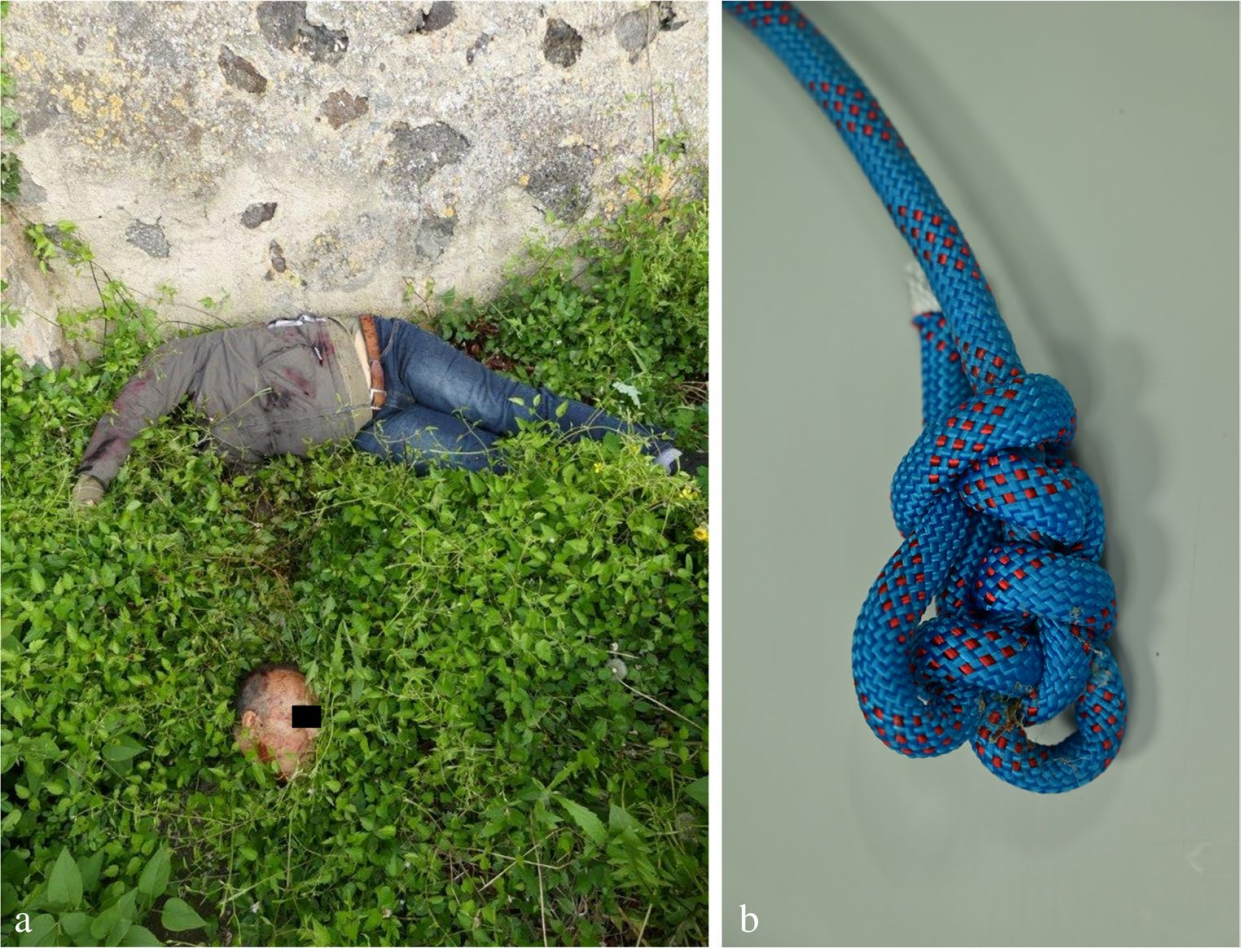
(**a**) Complete decapitation in a suicide who jumped from a stone wall with a hanging noose around his neck. (**b**) The formerly running noose is tightened at the position of the knot

A great number of hanging-induced *decapitations* – both complete and incomplete – have been reported in the forensic literature. A recent review [19] included 22 relevant cases described in English speaking articles. The fall heights ranged from 1.5 to 18 m. The neck separation mostly took place at the level of the first three cervical vertebrae. The hanging mark from the noose does not necessarily coincide with the severance plane. The victim may show intimal tears of the carotids (Amussat’s sign) as well as Simon’s bleedings on the lumbar and lower thoracic spine [126]. The caudal parts of the cervical column often protrude over the separation plane of the neck’s soft tissues, which tend to shorten due to their inherent elasticity [23, 127, 128]. A conspicuous finding may be seen on the hanging device in cases of decapitation: If a slipknot tightens almost completely, it typically contains muscle tissue and other biological material from the neck (Fig. 16).

*Simon’s haemorrhages* are located beneath the anterior longitudinal ligament of the lumbar and lower thoracic spine at the same level as the intervertebral disks (cf. Figure 13b). In fatalities from hanging, the frequency amounts to about 37% [129]. The sign is not specific for hanging, as it also occurs in other traumatizations associated with elongation/overextension of the spinal column, especially in traffic accidents [5]. Simon’s bleedings are more common in young victims and in cases with free body suspension. Blood extravasations into the psoas muscle [42] are probably due to traction forces similar to those exerted on the spine. In the autopsy material of Adamski et al. [92], Simon’s sign was found in 61% of the youngest age group up to 18 years, but in only 17% of the victims aged over 70 years. Up to now, Simon’s sign is considered to indicate a vital origin at least in the absence of putrefactive changes.

Apart from the neck regions, *muscle haemorrhages* may be discovered in the back and auxiliary breathing muscles of hanged persons. According to Schulz et al. [130], blood extravasations of this type were present in about 30% of the cases examined; the overlying skin and subcutis did not show corresponding haematomas. The occurrence of muscle haemorrhages was explained by increased respiratory exertions and/or agonal seizures. The finding is not specific for hanging, as it may also be present in cases of drowning [131] and in some deaths from a natural cause [132].

In the recent past, special attention has been paid to haemorrhages of the *intestinal wall* [133–135]. The bleedings were considered as an additional finding possibly associated with hanging. Okazi et al. [135] found bowel wall haemorrhage in 11.6% of their study material comprising 138 cases of hanging. Schulz et al. [133] determined a quite similar frequency (12%). In the authors‘ view, the blood extravasations might be due to abdominal congestion during agony. Rectal wall haemorrhage was diagnosed in 4 out of 102 hanging cases [134] and must not be misinterpreted as an indication of sexual assault.

Some internal findings, which in former times have been correlated with asphyxial deaths, such as subserous petechial bleedings [136], venous blood congestion and a liquid state of cadaveric blood do not have any diagnostic value, as they are quite unspecific.

**Fig. 17:**
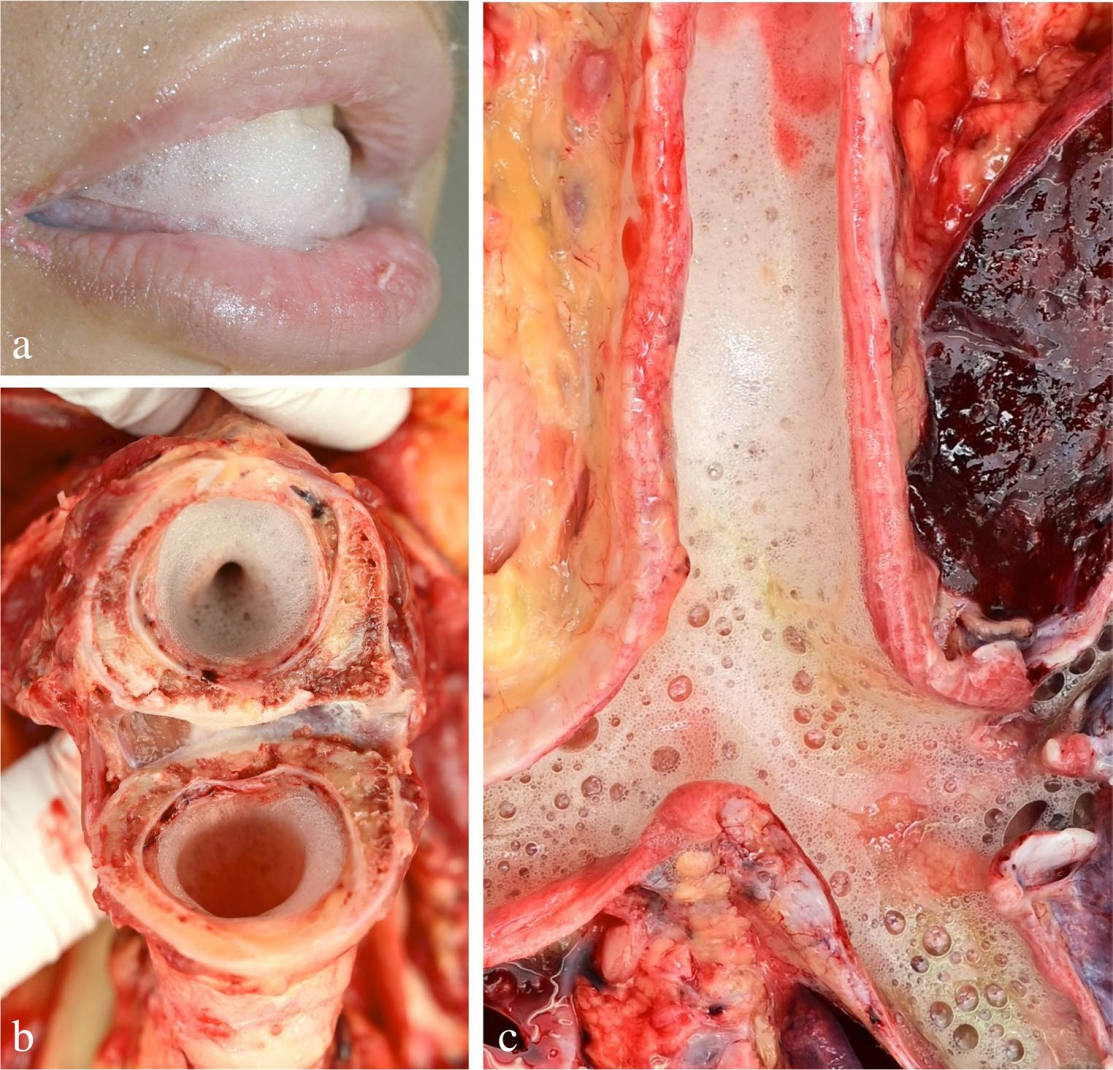
Whitish froth from pulmonary oedema in cases of hanging: foam coming from the mouth (**a**), occluding the larynx at the level of the cricoid cartilage (**b**) and in the trachea (**c**)

As far as hanging deaths are concerned, the *lungs* frequently show emphysematous changes (mainly located under the visceral pleura) and/or pulmonary oedema. The latter may be accompanied by whitish or pink froth filling the trachea and sometimes even exiting from mouth and nostrils, especially in hangings by frontal suspension with no or only minor pressure on the upper airways (Fig. 17) [85, 137]. Exceptionally, gastric contents may be aspirated into the deep airways [138, 139]. Older reports on the occurrence of hanging-induced cerebral air embolism could not be confirmed by subsequent studies [140, 141]. In contrast, it seems proven that in rare cases pre-existent venous thrombi may be detached during hanging so that they travel to the lungs and occlude pulmonary arteries [142].

In deaths from strangulation, the histological appearance of *lung tissue* was described as ‘haemorrhagic-dysoric syndrome’ being characterized by subpleural emphysema, dystelectases, haemorrhagic oedema and microembolism [143, 144]. Janssen [145] considered pulmonary giant cells to be pathognomonic for protracted asphyxia, but later investigations could not prove the assumed relationship [146, 147]. Up to now, the lung examination results as published in the pertinent literature do not seem to be consistent enough for proving a specific pathophysiology in hanging deaths.

For several years, research has focused on asphyxia-induced *inflammatory reactions* in lung tissue [148–151]. As far as hanging is concerned, it appears doubtful that the immune profile of the alveolar macrophages may undergo a significant change within the short agony following neck constriction. Nevertheless there are immunohistochemical studies suggesting that pulmonary mast cells may be activated even in cases of acute asphyxia [149]. In this context, elevated tryptase levels in blood might result from the degranulation of mast cells [150]. A great number of immunohistochemical studies have been conducted on asphyxial lungs targeting at parameters such as HIF1-alpha, TNF-alpha, IL-3, AQP5 and SP-A [148, 149, 151–155]. Regarding hypoxic brain damage, Barranco et al. [156] concluded that “immunohistochemistry has provided interesting and promising results, but further studies are needed in order to confirm and apply them in standard forensic practice”.

Already in the second half of the last century, many *biochemical parameters* were measured to distinguish between obstructive asphyxia and other causes of death. Prominent examples are lactate, free fatty acids, hypoxanthine, phospholipids, catecholamines, thyroglobulin, lactate dehydrogenase and guanidine. In 1990, a critical evaluation of the hitherto published data led to the conclusion that “the unambiguous diagnosis of obstructive asphyxia by biochemical analysis alone is not possible” [157].

*Forensic imaging* has been applied in order to verify fractures of the hyoid, the laryngeal cartilages and the cervical spine. Schulze et al. [110] described the presence of gas in the tissue adjacent to the laryngeal structures when fractures were present ('gas bubble sign') as a diagnostic indicator of neck trauma. On the other hand, Deininger-Czermak et al. [111] could find fracture-related gas bubbles only in a few cases of hanging. References in the literature concerning the incidence and frequency of cervical soft tissue emphysema and pneumomediastinum vary in a wide range (see [21]). In the thoracic and lumbar disks, centrally located gas accumulations were statistically correlated to complete hanging and younger ages (‘intervertebral vacuum phenomenon’) [158].

### Findings in primarily survived attempts of hanging

Survived hangings are predominantly due to suicide attempts and therefore seldom subject to forensic investigations. Hence, most reports dealing with this topic are found in the clinical literature (see [55]). The majority of victims are in a state of deep coma at first, often accompanied by cerebral seizures. Where there was initial cardiac arrest, severe neurologic sequelae have to be expected in a high percentage of the affected patients.

If death occurs after a latency period of several days, the consequences of global hypoxia are visible mainly on the brain tissue (cerebral oedema, multifocal encephalomalacia). In cases of pre-existing arteriosclerosis or thrombotic/embolic vascular occlusion, the cerebral lesions may be locally restricted [160]. In contrast to deaths in the initial phase of hanging (with the tightened noose still in place), intra- und subcutaneous bleeding under the hanging mark is common, if the victim was rescued at an early stage (before cardiac arrest) or if cardiovascular functions were restored through resuscitation measures [23, 159, 160].

## Discussion

The present article gives an overview of the main findings in fatal hangings. The primary focus is not on epidemiologic or other statistical data, but on the informative value of hanging-related injuries, especially with regard to the *manner of death*. From the criminalistic and medico-legal point of view, classification as suicide, accident or homicide is of crucial importance. As in many other fields of practical casework, mere knowledge of the pertinent literature cannot compensate for a lack of experience on the part of the examiner [27]. Moreover, it has to be stated that the frequencies of relevant findings as published in scientific papers often show an extremely wide margin (see [21, 161]).

In cases of hanging, the range of pitfalls and mistakes includes even the risk of overlooking signs indicating the cause of death, particularly if the body is no longer suspended and the noose has been removed [27, 54]. A great number of case reports demonstrate the high susceptibility to error when determining the manner of death. The dissimulation of a homicide by feigning a hanging suicide is especially challenging [25]. According to Hofmann and Haberda [26], the mere predominance of self-hangings can support the premature assumption of suicide.

In *homicidal acts* with suspension of the victim, hanging may be the actual way of committing the crime. Alternatively, the perpetrator can injure, poison or kill a person prior to the body’s suspension, mostly with the intention to simulate a suicide. As far as the first-mentioned case group is concerned, the noose is tightened during lifetime so that signs of vital hanging are to be expected. In the second group, the presence or absence of such signs depends on the latency between the initial harm and the secondary suspension.

In consideration of the hitherto published homicide cases simulating hanging suicides, it turns out that there is usually no significant delay between the primary assault and the subsequent hanging. This means that the victim’s cardiovascular and/or respiratory activity may still persist when the hanging noose tightens around the neck. Therefore, it would not be justified to exclude homicide just because autopsy reveals signs of vital suspension. It must also be borne in mind that in many cases of masked homicide the initial attack of the offender is directed against the neck, mostly by manual or ligature strangulation, which is why corresponding haematomas in the cervical region and petechial bleedings above the level of strangulation are common.

Medico-legal experts who survey a large number of hanging cases suppose a high dark figure of overlooked crimes. Second autopsies have shown that the thoroughness and the quality of the first post-mortem examination often leave much to be desired [22]. Even a comprehensive forensic autopsy does not necessarily prevent misinterpretation of findings leading to the incorrect assumption of suicidal hanging [162–165]. Already in the first half of the previous century, authors such as Böhmer [166] and Maync [167] emphasized the high risk that a homicide may go undetected when hanged victims are examined. Due to the realistic possibility of erroneous assessments, the reported number of homicidal hangings probably does not represent the true frequency of such crimes.

In 1984, Püschel et al. [168] recorded six homicidal hangings from the city of Hamburg and its surroundings. The authors estimated that the ratio of homicidal versus suicidal hangings is about 1:1000. In 2009, Sauvageau [169] found a remarkably high rate of homicidal hangings (1.6 per cent) in the Canadian province of Quebec. Physical superiority of the perpetrator or a pre-existing impairment of the victim caused for example by alcohol and/or drugs are factors which of course facilitate homicidal hanging. Nevertheless, thorough planning in combination with an element of surprise may be just as effective as mere physical overpowering [168, 170]. Relevant blood alcohol levels are frequently detected in homicidal hangings as well as in suicides [171].

In cases of *simulated self-hanging*, the suspicion of a crime can be based on a great variety of reasons including conspicuous circumstances and inconsistencies at the scene, e.g. the absence of a climbing aid (if necessary to reach the point of suspension), any drag marks on the floor and the presence of more than one groove on a wooden beam from repeated attempts to pull up the body. In addition, the clothing of the victim may indicate an offence prior to hanging (e.g. a topless female victim, underpants pushed down and wristband rolled on, garments put on back-to-front) [25].

**Fig. 18:**
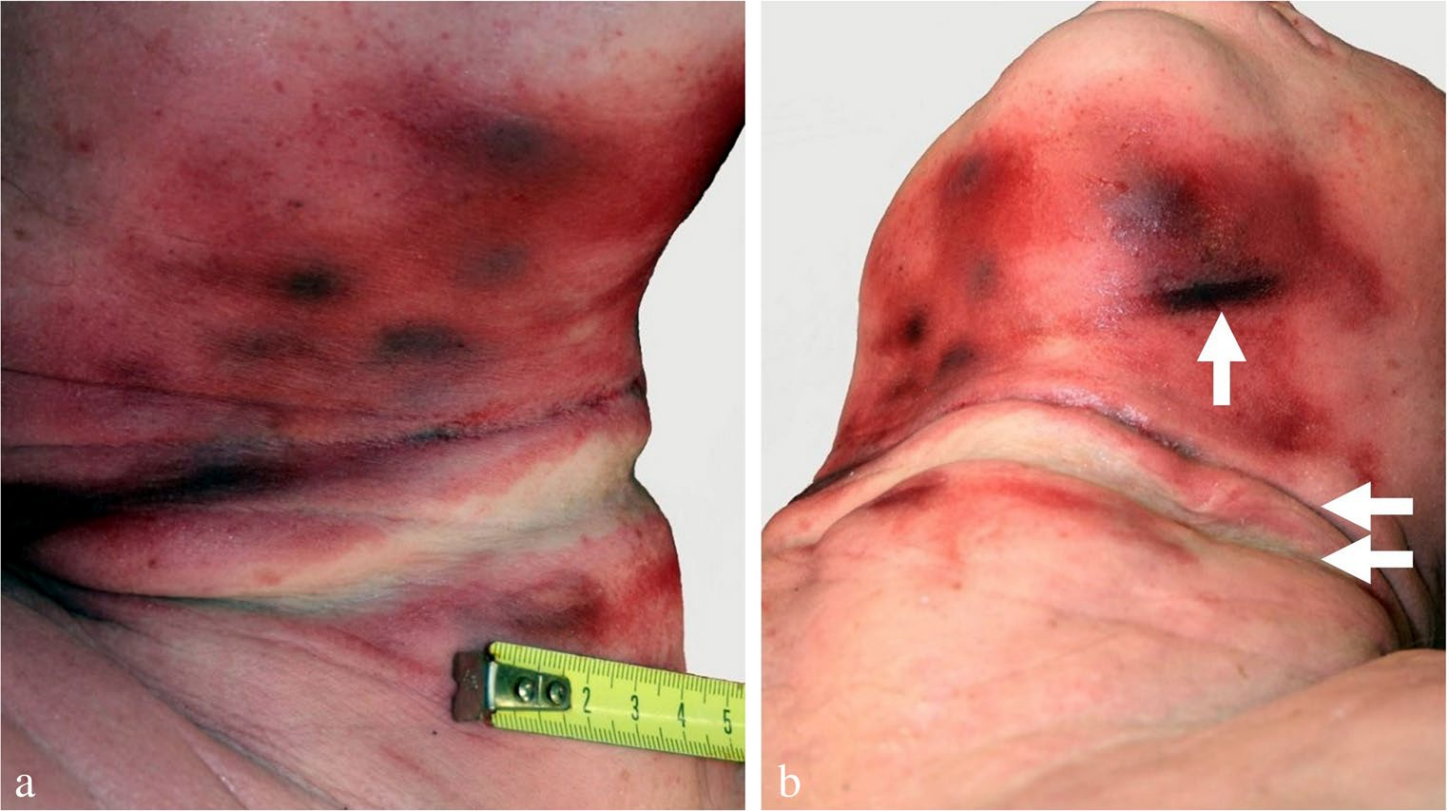
(**a**, **b**): Neck region of a 47-year-old female homicide victim killed by manual strangulation and then suspended to simulate suicide by hanging. Note the numerous and partly confluent bruises and the three hanging marks from repeated attempts to pull up the body (cf [164, 173])

Injuries not attributable to suicidal hanging should make the examiner think of foul play [25]: signs of manual or ligature strangulation (Fig. 18); more than one hanging mark although the noose has only one ligature turn; significant signs of supracervical blood congestion in cases of free suspension with the knot of the noose being located in the occipital region; haemorrhages in the anterior and lateral parts of the tongue due to compression between the dental arches; fracture of the cricoid cartilage; additional injuries in body regions apart from the neck (e.g. blunt head trauma, signs of smothering, bruises from gripping and overpowering, lesions from tying); drag marks from pulling the body along the ground. In the given context, it should be considered that convulsions as observed in filmed hangings [172] may cause minor impact injuries [10].

As outlined in this review, the published data on hanging-related findings are partly inconsistent with respect to their specificity and their relevance for proving suspension during life. From the viewpoint of crime detection, the most important item is particular attention to any conspicuous circumstances, features and injuries pointing to external interference. Awareness of potential errors, profound knowledge of the relevant literature and a thorough autopsy are crucially important requirements for a proper assessment of hanging cases. Mutual exchange of information between investigation officers and medico-legal experts including the death scene provides a holistic approach to clarify the facts and to determine the correct manner of death [68, 173].

## Conclusions

- From a forensic view, the main challenge in examining hanged victims is the reliable distinction between suicide, accident and homicide.
- Deaths from hanging may be accompanied by a multitude of morphological findings described in abundant textbooks and journal articles. However, it must be borne in mind that most of these signs are unspecific and that the frequency data vary considerably.
- The one-sided emphasis on ‘signs of vitality’ should be replaced by a comprehensive and integrated approach focusing on injuries which cannot be explained by suicidal or accidental hanging and therefore point to external influence. Vital signs may be present in homicides committed by primary hanging of the victim, but also in cases of secondary suspension subsequent to a different offence (e.g. manual or ligature strangulation, blunt traumatization or poisoning), so long as cardiorespiratory function persists.
- The following findings should not be regarded as proof of vital hanging, as they could be produced experimentally in post-mortem studies [28, 29]: ecchymotic skin ridges and peri-ligature blisters, muscle haemorrhages at the clavicular origin of the sternocleidomastoid, haematomas adjacent to laryngeal fractures, saliva traces exuding from the mouth, intimal tears of the carotid arteries.
